# A genome-first approach to variants in *MLXIPL* and their association with hepatic steatosis and plasma lipids

**DOI:** 10.1097/HC9.0000000000000427

**Published:** 2024-04-26

**Authors:** Leonida Hehl, Kate T. Creasy, Cecilia Vitali, Eleonora Scorletti, Katharina S. Seeling, Mara S. Vell, Miriam D. Rendel, Donna Conlon, Regeneron Genetics Center, Marijana Vujkovic, Inuk Zandvakili, Christian Trautwein, Kai M. Schneider, Daniel J. Rader, Carolin V. Schneider

**Affiliations:** 1 Department of Medicine III, Gastroenterology, Metabolic diseases and Intensive Care, University Hospital RWTH Aachen, Aachen, Germany; 2 Department of Genetics, Perelman School of Medicine, University of Pennsylvania, Philadelphia, Pennsylvania, USA; 3 Department of Medicine, Division of Translational Medicine and Human Genetics, Perelman School of Medicine, University of Pennsylvania, Philadelphia, Pennsylvania, USA; 4 The Institute for Translational Medicine and Therapeutics, The Perelman School of Medicine, University of Pennsylvania, Philadelphia, Pennsylvania, USA; 5 Regeneron Genetics Center, Tarrytown, New York, USA; 6 Department of Medicine, Division of Gastroenterology and Hepatology, Perelman School of Medicine, University of Pennsylvania, Philadelphia, Pennsylvania, USA; 7 Department of Internal Medicine, Division of Digestive Diseases, College of Medicine, University of Cincinnati, Cincinnati, OH, USA; 8 Department of Microbiology, Perelman School of Medicine, University of Pennsylvania, Philadelphia, Pennsylvania, USA

## Abstract

**Background:**

Common variants of the max-like protein X (MLX)-interacting protein-like (*MLXIPL*) gene, encoding the transcription factor carbohydrate-responsive element-binding protein, have been shown to be associated with plasma triglyceride levels. However, the role of these variants in steatotic liver disease (SLD) is unclear.

**Methods:**

We used a genome-first approach to analyze a variety of metabolic phenotypes and clinical outcomes associated with a common missense variant in *MLXIPL*, Gln241His, in 2 large biobanks: the UK Biobank and the Penn Medicine Biobank.

**Results:**

Carriers of *MLXIPL* Gln241His were associated with significantly lower serum levels of triglycerides, apolipoprotein-B, gamma-glutamyl transferase, and alkaline phosphatase. Additionally, *MLXIPL* Gln241His carriers were associated with significantly higher serum levels of HDL cholesterol and alanine aminotransferase. Carriers homozygous for *MLXIPL* Gln241His showed a higher risk of SLD in 2 unrelated cohorts. Carriers of *MLXIPL* Gln241His were especially more likely to be diagnosed with SLD if they were female, obese, and/or also carried the *PNPLA3* I148M variant. Furthermore, the heterozygous carriage of *MLXIPL* Gln241His was associated with significantly higher all-cause, liver-related, and cardiovascular mortality rates. Nuclear magnetic resonance metabolomics data indicated that carriage of *MLXIPL* Gln241His was significantly associated with lower serum levels of VLDL and increased serum levels of HDL cholesterol.

**Conclusions:**

Analyses of the *MLXIPL* Gln241His polymorphism showed a significant association with a higher risk of SLD diagnosis and elevated serum alanine aminotransferase as well as significantly lower serum triglycerides and apolipoprotein-B levels. *MLXIPL* might, therefore, be a potential pharmacological target for the treatment of SLD and hyperlipidemia, notably for patients at risk. More mechanistic studies are needed to better understand the role of *MLXIPL* Gln241His on lipid metabolism and steatosis development.

## INTRODUCTION

The increase in the prevalence of obesity, type 2 diabetes (T2D), and related metabolic and cardiometabolic comorbidities in recent years has led to a steady increase in steatotic liver disease (SLD) worldwide.^1–4^ The term SLD is used to describe a spectrum of liver diseases, ranging from isolated steatosis to metabolic dysfunction–associated steatohepatitis, liver fibrosis, and eventually resulting in cirrhosis or HCC.^2^ Isolated steatosis is characterized by the accumulation of lipid droplets in more than 5% of hepatocytes.^5^ The susceptibility and progression of SLD is highly influenced by a combination of different genetic and environmental factors.^6^ The strongest known risk factors for SLD are obesity, T2D, and metabolic syndrome, including hypertension and dyslipidemia.^6^

SLD is heritable and a recent large genome-wide association study identified over 70 genetic loci associated with SLD.^7,8^ One of the genome-wide significant variants reported is a coding variant in the max-like protein X (MLX)-interacting protein-like (*MLXIPL*) gene, namely Gln241His (rs3812316).^7^ This variant has an overall allele frequency of approximately 10% and has been associated with decreased plasma triglyceride (TG) levels.^7,9,10^ The *MLXIPL* gene encodes for the basic loop-helix-loop leucine zipper transcription factor named carbohydrate-responsive element-binding protein (ChREBP).^11^ ChREBP is part of the Myc/Max/Mad superfamily of transcription factors that regulates multiple physiological functions, including cellular proliferation, differentiation, and apoptosis.^11^ ChREBP activity depends on interaction with Max-like protein X (MLX), another member of the Myc/Max family, to form the heterotetrametric ChREBP/Mlx complex.^12^ The ChREBP/Mlx complex is highly active in hepatocytes in response to glucose and binds the carbohydrate response element motifs in the promoter region of target genes and transcriptionally activates expression of lipogenic genes, including L-type pyruvate kinase gene and fatty acid synthase.^12^ Consequently, ChREBP is an integral link between glucose sensing and lipid synthesis in the liver.^12,13^ *MLXIPL* Gln241His has never been studied for its effects on the function of ChREBP. In this study, we took a genome-first approach to analyzing liver and plasma lipid phenotypes associated with *MLXIPL* Gln241His.

## METHODS

### Resource availability

#### Lead contact

Further information and requests should be directed to and will be fulfilled by the Lead Contact Dr. med. Carolin V. Schneider (cschneider@ukaachen.de">cschneider@ukaachen.de).

#### Materials availability

This study did not generate new unique reagents.

#### Data and code availability

The data sets used in the current study have not been deposited in a public repository but are available when registered and approved at https://www.ukbiobank.ac.uk for UK Biobank (UKB).

### Experimental model and subject details and method details

#### UK biobank participants

**FIGURE 1:**
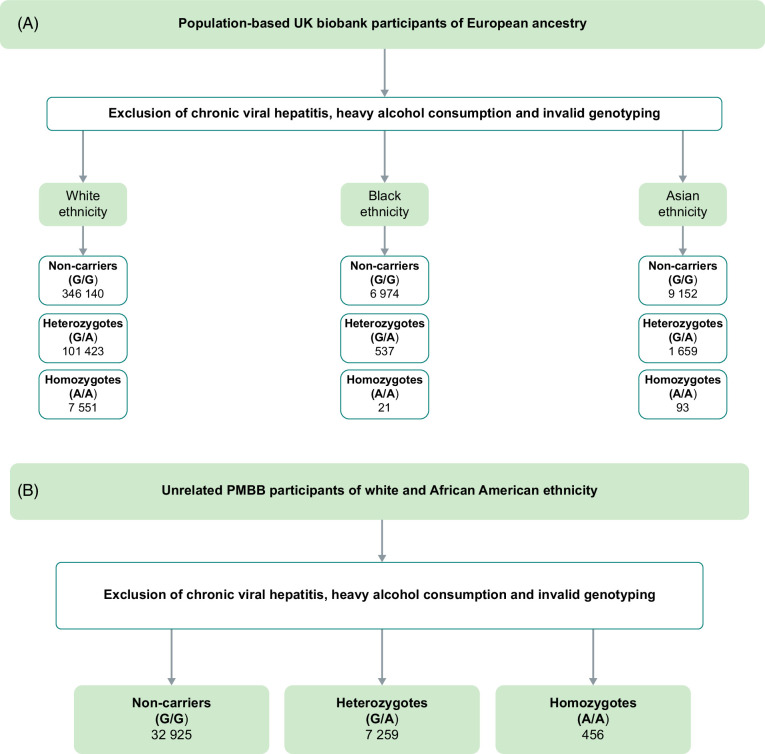
Overview of the analyzed cohorts. (A) UK Biobank (UKB) participants of European ancestry aged 37–73 years (*ML*XIPL rs3812316). (B) PMBB participants of 55% African American ancestry aged 25–105 years (*MLXIPL* Gln241His). Abbreviation: PMBB, Penn Medicine BioBank.

From 2006 to 2010, a population-based cohort research, the “UKB,” took place in the United Kingdom. It enrolled 502,511 participants aged 37–73 years old at baseline (Figure 1A).^14^ All participants were registered with the National Health Service of the United Kingdom and underwent an initial assessment, which was then followed by a continuous long-term follow-up (Supplemental Table S1, http://links.lww.com/HC9/A866). Blood samples were gathered, and physical measurements were acquired throughout every evaluated visit. All the participants gave their informed consent to be genotyped and have their data linked to medical records. A total of 360,141 White subjects were genotyped for the *MLXIPL* Ala258Val (rs35332062) gene polymorphisms, and for the *MLXIPL* Gln241His (rs3812316) gene polymorphisms, a total of 460,413 White subjects were genotyped. Participants with chronic hepatitis B or C (n=809) or self-reported excessive alcohol intake (>60 g alcohol per day for men and >40 g alcohol per day for women,^15^ n=4490) were excluded from the study. Our *MLXIPL* rs35332062 cohort includes 354,842 White participants with 101,001 heterozygous and 7451 homozygous carriers. To determine the diagnosis, patients with metabolic dysfunction–associated steatotic liver disease with steatosis and at least 1 cardiometabolic risk factor were considered according to the 2023 defined diagnostic criteria (Table 2).^4^

In addition, groups of 7263 (*MLXIPL* rs35332062) and 7532 (*MLXIPL* rs3812316) Black British and 10,904 (*MLXIPL* rs35332062) and 10,904 (MLXIPL rs3812316) Asian UKB subjects who did not have chronic hepatitis B or C nor self-reported excessive alcohol consumption but with available *MLXIPL* rs35332062 and *MLXIPL* rs3812316 genotyping were studied (Supplemental Tables S5–8, http://links.lww.com/HC9/A866, 15, 16, http://links.lww.com/HC9/A866 and Supplemental Figures 3–6, http://links.lww.com/HC9/A866 and Figure 1A).

Inpatient hospital records from 1996 to 2018 were used to determine diagnoses using the ICD-10 codes for all groups. The presence of the primary ICD-10 codes listed below was evaluated: overall liver disease (K70-K76), alcohol-associated liver disease (K70), toxic liver disease (K71), hepatic failure (K72), chronic hepatitis (K73), fibrosis and cirrhosis (K74), inflammatory liver diseases (K75), metabolic dysfunction–associated steatohepatitis (K75.8), other liver diseases (K76), SLD (K76.0), and malignant neoplasm of the liver and/or intrahepatic bile duct (C22). Through links to national death registries, the UKB receives death notifications (age at death and principal ICD diagnosis that led to death). Death or the end of hospital inpatient data collection in June 2021 was defined as the end of follow-up. Cardiovascular disorders (I00-I99) and liver diseases (K70-K77 and C22) were the leading specific causes of death. The UKB Access Committee has accepted the study (Project #71300).

#### Genetics

In the UKB, genome-wide genetic data and analyses of 488,000 participants were available. For the genotype data, the Haplotype Reference Consortium and UK10K were used. Genotyping of the *MLXIPL* Gln241His variant was available in 111,284 White UKB participants and in 111,226 White UKB participants for the *MLXIPL* rs35332062 variant.

#### Metabolomics

**FIGURE 2:**
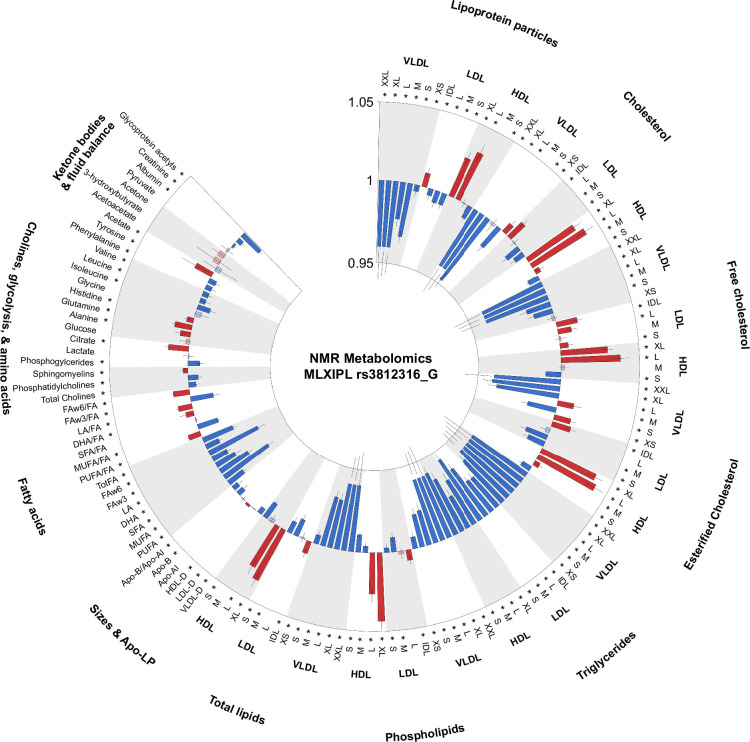
Circle plot for lipidomic analysis of white homozygous *MLXIPL* rs3812316 carriers in the UKB. Associations of metabolic biomarkers of homozygous *MLXIPL* rs3812316 carriers in White UKB participants. HRs (with 95% CIs) are presented per 1-SD higher metabolic biomarker on the natural log scale, stratified by age, sex, body mass index, and PC1-10. *False discovery rate–controlled *p*<0.01. Positive associations are displayed in red, while negative associations are blue (original code by Diego J. Aguilar-Ramirez). Abbreviations: DHA, docosahexaenoic acid; FAw3, omega-3 fatty acid; FAw6, omega-6 fatty acid; HDL-D, high-density lipoprotein particle diameter; LA, linoleic acid; LDL-D, low-density lipoprotein particle diameter; LP, lipoprotein; MUFA, monounsaturated fatty acids; PUFA, polyunsaturated fatty acids; SFA, saturated fatty acids; VLDL-D, very low-density lipoprotein particle diameter.

In a subgroup (n=105,348) of UKB participants, nuclear magnetic resonance–based metabolomic profiling was performed, and information on *MLXIPL* variants, Gln241His (Figure 2 and Supplemental Figure 1, http://links.lww.com/HC9/A866), and the nonsynonymous single-nucleotide variant rs139543215 were available (Supplemental Figure S7, http://links.lww.com/HC9/A866).^16^

#### Liver MRI proton density fat fraction

In a UKB subgroup of 40,453 participants, data of MRI estimated proton density fat fraction were available for the analysis of liver fat. Data of 8442 heterozygous *MLXIPL* Gln241His carriers and 585 homozygous *MLXIPL* Gln241His carriers were used (Table 1).

**TABLE 1:** Characteristics of *MLXIPL* rs3812316 homozygous or heterozygous carriers compared with noncarriers in UK Biobank

	Non carriers (G/G) n=346 140	Heterozygotes (A/G) n=101 423	Homozygotes (A/A) n=7 551	*p* G/G vs. A/G	*p* G/G vs. A/A
Characteristics	—	—	—	Univariate	Univariate
Age (y)	56.78±8.03	56.79±8.02	56.73±8.03	0.93	0.56
Sex (female), N (%)	188,037 (54.32)	55,162 (54.39)	4146 (54.91)	0.72	0.32
BMI (kg/m^2^)	27.38±4.77	27.44±4.78	27.53±4.88	**0.000088**	**0.006**
Alcohol (g/d)	9.04±10.12	9.25±10.28	9.53±10.42	**2.7E-9**	**0.000047**
Diabetes mellitus (%)	0.05±0.21	0.05±0.22	0.05±0.22	0.05	0.25
Liver fat (%)	4.88±4.92	4.94±4.84	5.09±5.14	0.35	0.3
Ethnicity (% White)	100	100	100		
Frequency of well-known SLD influencing genes^a^	—	—	—	Multivariate	Multivariate
*HSD17B13* rs72621367:TA	0.55±0.63	0.55±0.63	0.55±0.63	0.48	0.98
*PNPLA3* rs738409:G	0.43±0.58	0.43±0.58	0.43±0.58	0.34	0.73
Liver status					
ALT (U/L)	23.35±13.8	23.82±14.48	24.12±14.76	**7.7E-18**	**0.000012**
AST (U/L)	26.13±10.03	26.14±11.17	25.91±9.77	0.79	**0.046**
GGT (U/L)	37.31±41.39	35.90±39.85	34.29±41.50	**2.4E-26**	**6.2E-12**
Bilirubin (mg/dL)	0.53±0.26	0.53±0.26	0.54±0.28	**0.037**	**0.000488**
AP (U/L)	83.76±26.49	83±25.88	81.92±24.58	**1.1E-19**	**5.5E-11**
Lipid metabolism					
Triglycerides (mg/dL)	157.52±92.04	146.9±84.96	135.4±75.22	**1.4E-279**	**7.3E-111**
HDL cholesterol (mg/dL)	56.07±14.69	56.46±14.69	57.23±15.08	**1.5E-32**	**4.6E-20**
LDL cholesterol (mg/dL)	138.05±33.64	138.05±33.64	138.05±33.26	0.89	0.62
Cholesterol (mg/dL)	220.81±44.08	220.42±44.08	220.42±43.7	**0.013**	0.45
Apolipoprotein A1 (g/L)	1.54±0.27	1.54±0.27	1.54±0.27	0.67	0.77
Apolipoprotein-B (g/L)	1.04±0.24	1.03±0.24	1.03±0.23	**1.1E-10**	**0.000145**
Additional serum parameters					
Glucose	5.11±1.20	5.13±1.24	5.12±1.24	**0.007**	0.68
HbA1c (mmol/mol)	35.94±6.47	36.05±6.7	36.11±6.41	**0.000022**	**0.039**
IGF-1 (mmol/L)	21.37±5.67	21.53±5.67	21.79±5.86	0.48	0.98
** **Urate (μmol/L)	309.58±80.48	306.55±79.59	301.23±77.93	0.34	0.73

*Note:* Quantitative measures are expressed as means and SDs or as relative frequencies (%). All multivariable analyses were adjusted for age, sex, BMI, and PC1-10.

a 0=noncarrier, 1=heterozygous, 2=homozygous.

All significant p-values (<0.05) in bold.

Abbreviations: AP, alkaline phosphatase; GGT, gamma-glutamyl transferase; HbA1c, hemoglobin A1; SLD, steatotic liver disease.

#### MLXIPL nonsynonymous single nucleotide and predicted loss of function variant

To examine whether *MLXIPL* Gln241His may be a possible loss-of-function variant, we compared our results of the *MLXIPL* Gln241His polymorphism to a nonsynonymous single variant of *MLXIPL*, Arg651His (rs139543215) in the UKB. *MLXIPL* Arg651His does cause an amino acid change in *MLXIPL* and is described as “deleterious“ by the in silico tool SIFT and as “probably damaging“ by Polyphen. The same exclusion criteria as mentioned in the abstract “UKB participants” were used, and 13 carriers were analyzed in UKB (Table 4 and Supplemental Figure S7, http://links.lww.com/HC9/A866). *MLXIPL* rs1436362537 is a predicted loss of function (pLoF) variant. The same exclusion criteria as mentioned above were used, and 4 carriers were analyzed in a subgroup of White UKB participants (Supplemental Table S7, http://links.lww.com/HC9/A866).

#### Phenome-wide association study

A phenome-wide association study (PheWAS) was performed to examine *MLXIPL* Gln241His (Supplemental Table S2, http://links.lww.com/HC9/A866 and Supplemental Figure S2, http://links.lww.com/HC9/A866) and *MLXIPL* rs35332062 (not shown), and clinical outcomes, adjusted for age, sex, BMI, and principal components of ancestry 1-10. ICD-10 codes from electronic health records (EHRs) were collated throughout the study period, and duplicates were removed for each study subject. ICD-10 codes were converted to n=1625 associated Phecodes using the PheWAS package version 0.99.5-5 in R. For PheWAS analyses, Bonferroni correction was performed to account for multiple testing for the number of the major Phecode categories analyzed (*p*<0.05/1625).

#### Penn Medicine BioBank participants

The Penn Medicine BioBank (PMBB) recruited participants from across the University of Pennsylvania Health System’s clinical practice sites. Participants gave their informed consent for EHR data to be accessed. The Regeneron Genetics Center generated whole exome sequences from DNA isolated from preserved buffy coats and linked them to the Genome Reference Consortium Build 38. We excluded samples with low exome sequencing coverage (ie, less than 75% of targeted bases achieving 20×coverage; n=9), dissimilar reported and genetically determined sex (n=457), and genetic evidence of sample duplication (n=1137). ICD-9 and ICD-10 diagnostic codes were retrieved for the PMBB cohort. In all, 81,280 unrelated participants weregenotyped for the *MLXIPL* rs35332062 (n=40,640) and *MLXIPL* Gln241His (n=40,640; Figure 1B and Supplemental Figure S5B, http://links.lww.com/HC9/A866). Participants with chronic hepatitis B or C (n=1214) were excluded previously, including as well as alcohol-associated liver disease (571.0, K70.0), alcoholic hepatitis (571.1, K70.1), alcohol-associated fibrosis and sclerosis of the liver (571.2, K70.3), alcohol-associated cirrhosis of liver and/or ascites (571.2, K70.2), alcohol-associated hepatic failure, coma, and unspecified alcohol-associated liver disease (571.3, K70.4, K70.40, K70.41, K70.9), and alcohol dependence (303.0, 303.9, F10.229, F10.20) and participants with ethnicity listed as other or missing ethnicity (n=901). PMBB did not have data on daily alcohol usage. Between the baseline assessment and July 2020, ongoing hospital and outpatient records were analyzed to determine diagnoses using ICD-10 codes. We used the same ICD-10 diagnoses as in our UKB cohort. Furthermore, serum parameters for subjects were extracted from the time of registration in the PMBB through June 1, 2020; if several measurements were available, we chose the median value. Moreover, age, sex, BMI, and diabetes values were extracted from the EHR. Death notifications (defined as age at death and primary ICD code that led to death) were received by the PMBB through linkage to the EHR. Death or end of inpatient data collection at the end of May 2020 represents the end of follow-up. Cardiovascular disorders (I00-I99) and liver diseases (K70-K77 and C22) were the leading specific causes of death.

#### Quantification and statistical analysis

Unpaired, two-tailed tests or the Mann-Whitney *U* test were used to analyze all continuous variables, as well as a suitable multivariable model. The data are presented as a mean±SD (normal distribution). The Chi-square test was used to evaluate the contingency tables for all categorical variables that are displayed as relative (%) frequencies. The 95% CIs for the ORs/HRs are indicated in brackets. Cox proportional hazard regression models were used to calculate HRs. To test for any independent associations, multivariable logistic regressions were performed. Age, sex, BMI, and principal components of ancestry 1–10 were used to adjust all performed multivariable analyses. In this association study, participants without the specific ICD-10 code are the reference groups for analyzing any specific ICD-10 code.

If *p*<0.05, differences were considered statistically significant. The following programs were used to analyze our collected data: R version 4.0.2 (R Foundation for Statistical Computing; Vienna, Austria), SPSS Statistics version 26 (IBM; Armonk, NY, USA) and Prism version 8 (GraphPad, LaJolla, CA). To create the graphical abstract, BioRender was used.

## RESULTS

### Study population

To assess the effects of *MLXIPL* Gln241His on SLD-related traits, we applied a genome-first approach in both the UKB and the PMBB (Figure 1). Exome sequencing data available for all UKB participants analyzed for missense variants in *MLXIPL*. The UKB study includes 451,444 White participants, with 22.5% being heterozygous and 1.7% being homozygous for *MLXIPL* Gln241His (Table 1); in the PMBB, 17.9% were heterozygous, and 1.1% of White participants were homozygous for Gln241His (Table 3). The clinical characteristics of *MLXIPL* Gln241His genotype in the UKB are shown in Tables 1 and 2.

**TABLE 2:** Characteristics of *MLXIPL* rs3812316 homozygous or heterozygous carriers compared with noncarriers in UKB

	Noncarriers (G/G) n=346 140	Heterozygotes (A/G) n=101 423	Homozygotes (A/A) n=7 551	*p* G/G vs. A/G	*p* G/G vs A/A	*p* G/G vs. A/G	*p* G/G vs. A/A	aOR G/G vs. A/G	aOR G/G vs. A/A
Liver status				Univariate	Univariate	Multivariate	Multivariate	Multivariate	Multivariate
ALT ≥ULN, N (%)	21,790 (6.3)	7014 (6.92)	566 (7.5)	**8E-13**	**0.00003**	**4.8E-11**	**0.0005**	1.11 [1.08–1.14]	1.21 [1.11–1.31]
AST ≥ULN, N (%)	15,323 (4.43)	4445 (4.38)	329 (4.36)	0.58	0.73	0.34	0.40	0.990	0.981
ICD10 coded diagnoses									
Alcohol-associated liver disease (K70), N (%)	1039 (0.30)	325 (0.32)	30 (0.4)	0.30	0.13	0.44	0.17	1.068	1.325
Toxic liver disease (K71)	104 (0.03)	19 (0.02)	2 (0.03)	0.06	0.86	0.06	0.84	0.623	0.882
Hepatic failure (K72)	584 (0.17)	199 (0.2)	19 (0.25)	0.07	0.08	0.11	0.09	1.163	1.493
Chronic hepatitis (K73)	144 (0.04)	36 (0.04)	4 (0.05)	0.39	0.63	0.39	0.65	0.853	1.273
Fibrosis and cirrhosis (K74)	1.368 (0.4)	367 (0.36)	30 (0.4)	0.13	0.98	0.09	0.89	0.915	1.005
Inflammatory liver diseases (K75)	1.101 (0.32)	352 (0.35)	22 (0.29)	0.15	0.68	0.21	0.60	1.091	0.916
MASH (K75.8)	405 (0.12)	123 (0.12)	7 (0.09)	0.73	0.54	0.83	0.45	1.037	0.792
Other liver diseases (K76)	6.948 (2.01)	2142 (2.11)	166 (2.2)	**0.038**	0.24	0.05	0.33	1.053	1.097
SLD (K76.0)	3.749 (1.08)	1117 (1.10)	101 (1.34)	0.62	**0.035**	0.64	0.07	1.017	1.238
Malignant neoplasm of the liver and/or bile duct (C22)	639 (0.18)	188 (0.19)	11 (0.15)	0.96	0.44	0.99	0.42	1.004	0.789
** **MASLD	3 749 (1.08)	1 117 (1.10)	101 (1.34)	0.62	**0.035**	0.64	0.07	1.016	1.099
Survival, N (%)									
All-cause mortality	23,943 (6.92)	7230 (7.13)	512 (6.78)	**0.020**	0.64	0.08	0.54	1.033	0.979
Liver-related death	713 (0.21)	254 (0.25)	18 (0.24)	**0.007**	0.54	**0.012**	0.60	1.22 [1.05–1.40]	1.158
Cardiovascular death	4798 (1.39)	1522 (1.50)	99 (1.31)	**0.007**	0.58	**0.023**	0.55	1.084 [1.02–1.15]	0.945

*Note:* Quantitative measures are expressed as number of participants (N) and as relative frequencies (%). All multivariable analyses were adjusted for age, sex, BMI, and PC1-10).

All significant p-values (<0.05) in bold.

Abbreviations: C22, malignant neoplasm of the liver and/or bile duct; MASH, metabolic dysfunction–associated steatohepatitis; MASLD, metabolic dysfunction–associated steatotic liver disease (patients diagnosed with NAFLD and at least one cardiometabolic risk factor); SLD, steatotic liver disease.

### MLXIPL Gln241His is positively associated with SLD-related traits

In White UKB participants, alanine aminotransferase levels, but not aspartate aminotransferase levels, were significantly higher in carriers of *MLXIPL* Gln241His compared to noncarriers (all *p*<0.0001, Table 1). Bilirubin levels were also significantly higher in Gln241His carriers (heterozygous vs. noncarriers *p*=0.037, homozygous vs. noncarriers *p*=0.0004888, Table 1). By contrast, carriers had significantly lower levels of gamma-glutamyl transferase (*p*<6.2×10^−12^, Table 1) and alkaline phosphatase than controls (*p*<5.5×10^−11^, Table 1). Analyses of liver fat percentage measured by MRI showed a higher liver fat percentage in homozygous and heterozygous *MLXIPL* Gln241His carriers compared to noncarriers but unfortunately did not meet statistical significance due to too small sample size (Table 1). In the UKB and PMBB interaction, analysis with BMI and BMI≥30 kg/m^2^ described a statistically significant positive association between *MLXIPL* Gln241His carriage and higher BMI (Supplemental Tables S13–S16, http://links.lww.com/HC9/A866). Homozygotes for *MLXIPL* Gln241His had a significantly greater risk of a SLD diagnosis; heterozygous carriers of *MLXIPL* Gln241His had a higher risk of liver disease (ICD10 code K76) compared to controls (*p*-value=0.038, Table 2) that remained significant after multivariate analysis. Additional analyses of participants with diagnosed hepatic steatosis and at least 1 of 5 metabolic dysfunction–associated steatotic liver disease (MASLD)-defining cardiovascular risk factors showed a significantly higher risk for metabolic dysfunction–associated steatotic liver disease in homozygous *MLXIPL* Gln241His carriers (Table 2 and Supplemental Table S12, http://links.lww.com/HC9/A866). UKB subgroups of Black or Asian participants showed similar trends in carriers of *MLXIPL* Gln241His (Supplemental Tables S3–S6, http://links.lww.com/HC9/A866 and Supplemental Figures S3–S5, http://links.lww.com/HC9/A866). In Black participants, homozygous carriers of *MLXIPL* Gln241His had a significantly higher risk of liver disease while heterozygous carriers showed a higher risk of malignant neoplasm of the liver and/or bile duct (adjusted odds ratio [aOR] 116.183 [95% CI: 11.586–1165.033]; aOR 5.210 [95% CI: 1.009–26.919], Supplemental Table S4, http://links.lww.com/HC9/A866). Asian heterozygous *MLXIPL* Gln241His carriers had a higher risk of alcohol-associated liver disease (aOR 2.931 [95% CI: 1.304–6.586], Supplemental Table S6, http://links.lww.com/HC9/A866). In UKB, compared to noncarriers, heterozygous carriers of the *MLXIPL* Gln241His had significantly higher liver-related mortality rates (liver-related mortality aOR 1.22 [95% CI: 1.05–1.40]; Table 2).

Analyses of PMBB participants replicated these findings. There was a significant increase in alanine aminotransferase in *MLXIPL* Gln241His homozygous carriers compared to controls (*p*=0.013, Table 3). Additionally, homozygous carriers had a significantly higher risk of liver diseases, including SLD (*p*=0.012, aOR=1.578 [95% CI: 1.097–2.268]; Table 3).

**TABLE 3:** Baseline characteristics of homozygous (A/A) or heterozygous *MLXIPL* rs3812316 carriers (A/G) compared with noncarriers (G/G) in PMBB

	Noncarriers (G/G) n=32 925	Heterozygotes (G/A) n=7 259	Homozygotes (A/A) n=456	*p* G/G vs. G/A	*p* G/G vs. A/A
Characteristics	—	—	—	Univariate	Univariate
Age (y)	54.90±16.75	56.15±16.53	57.20±16.73	**7.4E-9**	**0.004**
Women (%)	35.9	35.9	35.9	—	—
BMI (kg/m^2^)	29.54±7.30	28.72±6.54	29.03±7.09	**1.5E-19**	0.15
Diabetes mellitus (%)	0.25±0.43	0.24±0.43	0.27±0.44	0.40	0.75
Ethnicity (% White)	47.9	47.9	47.9	—	—
Frequency of well-known SLD influencing genes^a^	—	—	—	Multivariate	Multivariate
*HSD17B13* rs72621367:TA	0.29±0.51	0.42±0.59	0.47±0.63	0.09	0.84
*PNPLA3* rs738409:G	0.43±0.59	0.44±0.59	0.45±0.60	**0.022**	0.50
Liver profile					
ALT (U/L)	24.51±41.90	25.87±48.44	30.22±56.77	0.09	**0.013**
ALT ≥ULN (%)	1 242 (3.77)	292 (4.02)	24 (5.26)	0.69	0.13
AST (U/L)	24.72±39.51	25.35±38.82	28.69±38.20	0.74	0.10
AST ≥ULN (%)	917 (2.79)	194 (2.67)	21 (4.61)	0.42	**0.040**
Lipid profile					
Triglycerides (mg/dL)	122.24±86.81	119.54±88.77	110.16±63.05	**0.00008**	**0.001**
HDL cholesterol (mg/dL)	50.28±15.32	50.18±16.23	47.17±14.83	0.95	0.42
** **LDL cholesterol (mg/dL)	99.23±32.60	98.50±32.33	98.79±32.6	0.58	0.68
** **Cholesterol (mg/dL)	175.30±39.64	174.19±39.40	172.00±38.21	0.17	0.19
ICD10 coded diagnoses					
Fibrosis and cirrhosis (K74)	157 (0.48)	47 (0.65)	3 (0.66)	0.07	0.61
Inflammatory liver diseases (K75)	661 (2.01)	138 (1.90)	9 (1.97)	0.71	0.93
MASH (K75.8)	753 (2.29)	147 (2.03)	10 (2.19)	0.15	0.77
Other liver diseases (K76)	1 503 (4.56)	320 (4.41)	32 (7.02)	0.86	**0.012** ^a^
** **SLD (K76.0)	1 102 (3.35)	234 (3.22)	23 (5.04)	0.90	0.06

*Note:* Quantitative measures are expressed as means and SDs or as relative frequencies (%). All multivariable analyses were adjusted for age, sex, BMI, and PC1-4.

All significant p-values (<0.05) in bold.

a 0=non carrier, 1=heterozygous, 2=homozygous; aOR=1.578 [1.097–2.268].

Abbreviations: GGT, gamma-glutamyl transferase; MASH, metabolic dysfunction–associated steatohepatitis; SLD, steatotic liver disease.

**FIGURE 3:**
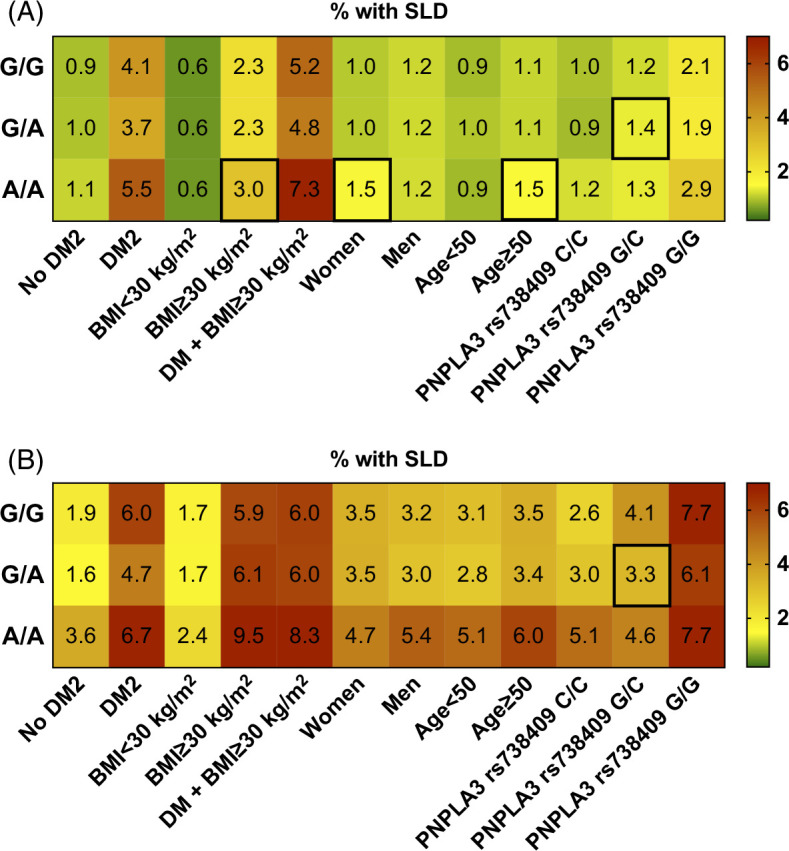
Frequency of SLD for *MLXIPL* rs3812316 genotype among various subgroups in the (A) UKB and (B) PMBB. Frequency of SLD for *MLXIPL* Gln241His genotype among various subgroups in the (A) UKB and (B) PMBB. Relative frequencies (%) are shown and visualized by color coding (right panel). Significant outcomes are outlined in black. Abbreviations: DM2, diabetes mellitus type 2; PNPLA3, patatin-like phospholipase domain-containing protein 3; SLD, steatotic liver disease.

The association of *MLXIPL* Gln241His with SLD was more pronounced in subgroups with common SLD comorbidities and risk factors like T2D or obesity, as well as carriers of patatin-like phospholipase domain-containing protein 3 (*PNPLA3*) I148M (Figure 3). Homozygote female carriers of *MLXIPL* Gln241His had a significantly increased frequency of SLD in comparison to noncarriers, whereas homozygote male carriers did not show such an effect (1.0% vs. 1.5%, Figure 3).

### MLXIPL Gln241His is associated with lower plasma TGs and TG-rich lipoproteins

Given that hepatic steatosis represents excess accumulation of lipid in hepatocytes that could be due to impaired TG secretion, it is of great interest to examine the effect of SLD-associated genetic variants on plasma lipid and lipoprotein traits. In UKB, the lipid profile in homozygotes for *MLXIPL* Gln241His was characterized by lower plasma TG (*p*<7.3×10^−111^) and apolipoprotein-B (*p*=0.000145) and higher plasma HDL cholesterol levels (and *p*<4.6×10^−20^) (Table 1). The UKB provides data on 168 serum metabolites measured by nuclear magnetic resonance in a subset of participants (n=105,348). We analyzed these data among *MLXIPL* Gln241His variant carriers and found that homozygous carriers had fewer and smaller VLDL particles, consistent with the reduced TG levels (Figure 2). Heterozygous carriers had significantly lower phospholipids in medium VLDL and significantly smaller HDL particles in comparison to large HDL, which had significantly more phospholipids (Supplemental Figure S1, http://links.lww.com/HC9/A866). In PMBB, there was a significant reduction in TG levels in heterozygous as well as homozygous carriers (heterozygous 119.5±88.8, homozygous 110.2±63.1 mg/dL vs. noncarriers 122.2±86.8 mg/dL; *p*<0.001) with no change in HDL cholesterol or LDL-C (Table 3).

### Obesity and other phenotypes

We performed a PheWAS to identify clinical phenotypes associated with *MLXIPL* Gln241His and corrected for age, sex, BMI, and principal components 1-10 (Supplemental Figure S2, http://links.lww.com/HC9/A866). We associated 2 Bonferroni-significant phenotypes with *MLXIPL* Gln241His in UKB (Supplemental Table S2, http://links.lww.com/HC9/A866). The strongest association has reduced the risk of gout (OR=0.8; *p*=5.64×10^−12^, Supplemental Table S2, http://links.lww.com/HC9/A866). In comparison to noncarriers, heterozygous and homozygous *MLXIPL* Gln241His carriers had significantly decreased plasma urate levels (Table 1). Heterozygous *MLXIPL* rs3812316 carriers had significantly increased serum glucose and hemoglobin A1 levels (Table 1).

### MLXIPL nonsynonymous single-nucleotide variant

We compared our results of *MLXIPL* Gln241His to a nonsynonymous single-nucleotide variant of MLXIPL, Arg651His (rs139543215), to determine whether the *MLXIPL* rs3812316 gene polymorphism might be a possible loss-of-function variant. *MLXIPL* Arg651His is described as deleterious by different in silico tools, for example, Polyphen and SIFT. We found 13 participants heterozygous for the rs139543215 *MLXIPL* variant in the UKB. Carriers of the rare *MLXIPL* Arg651His showed significantly higher serum levels of LDL cholesterol compared to noncarriers (mean serum levels 138.05 vs. 160.48, *p*=0.022; Table 4). In addition, we analyzed the data on 168 serum metabolites measured by nuclear magnetic resonance among heterozygous carriers of *MLXIPL* Arg651His variant in a subgroup of White UKB participants. Our analysis showed that heterozygous carriers had a higher concentration of phospholipids in VLDL, and the concentration of TGs was higher in LDL and small HDL (Supplemental Figure S7, http://links.lww.com/HC9/A866). In comparison to the carriers of *MLXIPL* Gln241His, heterozygous carriers of *MLXIPL* Arg651His were associated with higher concentrations of, for example, lipoprotein particles, cholesterol, free and esterified cholesterol, and total lipids in VLDL, but our results did not meet statistical significance (Supplemental Figure S7, http://links.lww.com/HC9/A866). Further analyses in larger cohorts of *MLXIPL* Arg651His variant carriers are necessary to evaluate its impact on different serum metabolites (Supplemental Figure S7, http://links.lww.com/HC9/A866). In addition, we analyzed a pLoF of *MLXIPL*, rs1436362537, which was associated with significantly higher apolipoprotein-B in White heterozygous carriers in a UKB subgroup (1.03±0.24 vs. 1.19±0.04, Supplemental Table S7, http://links.lww.com/HC9/A866). These results are consistent with the reduced function of *MLXIPL* leading to reduced expression of ChREBP and suggest that Gln241His does not represent a pLoF.

**TABLE 4:** Characteristics of heterozygous *MLXIPL* rs139543215 nonsynonymous single-nucleotide variant compared with rs3812316 noncarriers in a UKB subgroup of White participants

	Noncarriers (G/G) n=200,587	Heterozygotes (A/G) n=13	*p* G/G vs. A/G	*p* G/G vs. A/G
Characteristics	—	—	Univariate	Multivariate
Age (y)	56.72±8.03	56.70±10.41	0.99	0.93
BMI (kg/m^2^)	27.34±4.73	26.35±4.01	0.51	0.41
Alcohol (g/d)	9.09±10.05	9.01±5.46	0.98	0.52
Diabetes mellitus (%)	0.05±0.21	0.10±0.32	0.43	0.38
Ethnicity (% White)	100	100	—	—
Liver profile (U/L)				
ALT	23.37±13.73	22.63±7.4	0.86	0.65
AST	26.07±9.98	24.82±5.88	0.69	0.51
GGT	36.40±40.29	30.47±19.18	0.64	0.48
AP	83.32±25.66	84.20±21.47	0.91	0.76
Lipid profile				
Triglycerides (mg/dL)	153.99±89.39	175.23±69.92	0.46	0.49
HDL cholesterol (mg/dL)	56.46±14.69	52.59±13.53	0.42	0.62
LDL cholesterol (mg/dL)	138.05±33.26	160.48±25.52	0.35	**0.022**
Cholesterol (mg/dL)	221.19±44.08	244.01±36.74	0.1	0.05
Apolipoprotein A1 (g/L)	1.55±0.27	1.48±0.29	0.42	0.63
Apolipoprotein-B (g/L)	1.03±0.24	1.15±0.15	0.13	0.1
Urate (μmol/L)	307.66±79.82	330.21±103.11	0.37	0.70
ICD10 coded diagnoses, N (%)				
Alcohol-associated liver disease (K70)	488 ( 0.24)	0 (0)	0.87	0.99
Toxic liver disease (K71)	45 (0.02)	0 (0)	0.96	0.99
Hepatic failure (K72)	329 (0.16)	0 (0)	0.89	0.99
** **Chronic Hepatitis (K73)	78 (0.04)	0 (0)	0.95	0.99
Fibrosis and cirrhosis (K74)	667 (0.33)	0 (0)	0.85	0.99
Inflammatory liver diseases (K75)	599 (0.3)	0 (0)	0.86	0.99
MASH (K75.8)	215 (0.11)	0 (0)	0.91	0.99
SLD (K76.0)	1975 (0.98)	0 (0)	0.74	0.99

*Note:* Quantitative measures are expressed as means and SDs or as relative frequencies (%). All multivariable analyses were adjusted for age, sex, BMI, and PC1-4.

All significant p-values (<0.05) in bold.

Abbreviations: AP, alkaline phosphatase; GGT, gamma-glutamyl transferase; MASH, metabolic dysfunction–associated steatohepatitis; SLD, steatotic liver disease.

Our analyses of another interesting missense variant of *MLXIPL*, the *MLXIPL* rs35332062, Ala258Val, gene polymorphism, which is in very high linkage disequilibrium (0.97) with *MLXIPL* Gln241His and a possible haplotype,^17^ showed comparable results with regard to liver metabolism and SLD (Supplemental Tables S8–S11, http://links.lww.com/HC9/A866 and Supplemental Figures S4–S7, http://links.lww.com/HC9/A866). *MLXIPL* Ala258Val is a missense single-nucleotide polymorphism located in the coding region of *MLXIPL.*^7,17^

## DISCUSSION

We found that carriers of *MLXIPL* Gln241His had a higher risk of SLD diagnosis and higher serum levels of alanine aminotransferase accompanied by lower levels of TGs and apolipoprotein-B. These results suggest that *MLXIPL* Gln241His may result in reduced hepatic TG secretion, leading to an increased risk of steatosis. ChREBP is a large transcription factor that has a role in integrating hepatic carbohydrate and lipid metabolism.^18^ ChREBP contains multiple functional domains.^19^ The highly conserved N-terminal region contains the low-glucose inhibitory domain that regulates the transcriptional activation of the glucose-activated conserved element (GRACE) domain.^20^ The C-terminal region includes a polyproline-rich domain, a basic helix-loop-helix-zipper domain, and a dimerization and cytoplasmic localization domain, which are involved with the binding to co-factors and DNA.^20^ The dynamics of the functional regulation of ChREBP are only partially understood and likely involve several post-transcriptional modifications.^19^ Moreover, it is apparent that the N-terminal region of ChREBP, where Gln241His is located, plays a key role in modulating ChREBP transcriptional activity in response to nutrients. In the nucleus, ChREBP binds to carbohydrate response element motifs on glycolytic and lipogenic gene promotors and activates their expression.^21,22^ *Per se*, the substitution of Gln with His is predicted to be tolerated by most of the in silico algorithms. However, this variant is located within the highly conserved GRACE domain, which dynamically interacts with the low-glucose inhibitory domain to regulate the binding of ChREBP to nutrients and hormones and ultimately determine its subcellular localization and its transcriptional activity.^23^ It is possible that the Gln241His variant may alter the interaction between low-glucose inhibitory domain and GRACE or alter the transactivation ability of the GRACE domain.

Overexpression of ChREBP leads to increased TG levels in the liver through increased *de novo* lipogenesis.^18^ At the same time, overexpression of ChREBP increases plasma FGF21 levels and decreases plasma angiopoietin-like 3 levels, leading to lower plasma TGs through TG disposal in adipose tissue and oxidative tissue.^18,24,25^ In addition, rodent studies showed that hepatic deletion of ChREBP in mice led to reduced induction of glycolytic and lipogenic genes in response to nutrients, for example, sucrose.^26^ Liver TG levels were furthermore decreased in mice with liver-specific deletion of ChREBP.^26^ Further analyses of rare pLOF variants and parameters of the liver and lipid metabolism did not show statistical significant results of TG levels due to very small sample sizes. ChREBP is further known to play an important role in the development of T2D and to be associated with obesity.^27–29^ Our analyses showed a positive correlation between Gln241His carriers and higher BMI, which might indicate an increased risk of SLD development.

*MLXIPL* Gln241His is associated with significantly lower TG serum levels and a significantly higher BMI in White heterozygous and homozygous carriers in UKB. These findings could be replicated in our Asian subgroup. In the PMBB, the decrease in TG serum levels follows a gene-dose pattern (approximately 8% decrease in heterozygotes vs. 14% decrease in homozygotes). *MLXIPL* Gln241His could result in reduced secretion of VLDL-TGs from the liver. Further studies are required to investigate the exact underlying mechanisms.

In addition, our analyses indicated that *MLXIPL* also plays a role in uric acid metabolism. Studies revealed that uric acid is the result of fructose-induced fat accumulation in the liver, and it is associated with SLD severity and other factors related to the metabolic syndrome.^30^ Uric acid upregulates hepatic fructokinase, an enzyme catalyzing the phosphorylation of fructose,^30,31^ which in turn increases the lipogenic effects of fructose such as fat accumulation or greater oxidative stress.^32^ In other studies, urid acid has been shown to directly regulate hepatic lipogenesis through mitochondrial translocation of NADPH oxidase and led to increased de novo lipogenesis and TG in hepatocytes.^33,34^ Another study showed that ChREBP’s activity in fructose metabolism and its nuclear translocation can be blocked by allopurinol, a urate-lowering medication often prescribed to treat gout.^30^

In our study, *MLXIPL* Gln241His showed a trend toward lower urate serum levels in homozygous and heterozygous carriers compared to noncarriers in UKB. A study of Slovak women described a significant association between higher plasma urate serum levels and *MLXIPL* Gln241His.^35^ Moreover, our PheWAS analysis demonstrated that there was a reduced risk of gout in *MLXIPL* Gln241His carriers. All these results underline that *MLXIPL* Gln241His might be involved in early stages of SLD.

The effect of *MLXIPL* Gln241His on liver disease was most pronounced among those at the highest risk of SLD, namely those with obesity, T2D mellitus, and carriers of *PNPLA3* I148M. Interestingly, the effects of *MLXIPL* Gln241His were also more pronounced in women. This may be explained by reports that ChREBP is transcriptionally regulated by estrogen receptor signaling activity, although the exact mechanisms of this regulation and SLD prevalence are currently not well understood.^36,37^ Thus, the interactions and mechanisms of estrogen signaling, ChREBP activity, and SLD should be further explored.

One of our study limitations is that we used data that were previously collected from participants and provided by the UKB and PMBB. Therefore, we are aware that there might be different preanalytical and biological variables that could have a significant influence on TG serum levels that could have an impact on our results.^38^ Furthermore, there was only limited information on liver disease severity at baseline available since for instance in the UKB the evaluation of liver disease severity through noninvasive tests was included in the baseline assessment data collection.^39^ Additionally, power is challenged for participants homozygous for *MLXIPL* Gln241His due to relatively small numbers.

In conclusion, our study describes the inverse association of an *MLXIPL* coding variant with an increased risk of SLD but decreased plasma TG levels.

Our findings suggest that ChREBP influences the risk of hepatic steatosis and SLD by affecting the rate of hepatic VLDL-TG secretion. Further studies are necessary to investigate the impact of Gln241His on ChREBP function and the mechanisms associated with ChREBP, which are involved in the development of SLD.

## Supplementary Material

SUPPLEMENTARY MATERIAL
