# Supplementary material for: A genome-first approach to variants in MLXIPL and their association with hepatic steatosis and plasma lipids
Source: Hepatol Commun. 2024 Apr 26;8(5):e0427. doi: 10.1097/HC9.0000000000000427 (PMC12333743; doi:10.1097/HC9.0000000000000427)
Supplement: SUPPLEMENTARY MATERIAL [file hc9-8-e0427-s001.docx]

**Supplementary Data of
“A genome-first approach to variants in *MLXIPL* and their association with hepatic steatosis and plasma lipids“**

Table of contents

[Supplementary Table 1. Definitions of Outcomes and Covariates 3](#_Toc160546420)

[Supplementary Figure 1. Multivariable PheWAS for MLXIPL rs3812316 in UKB corrected for age, sex, BMI, and PC1-10 6](#_Toc160546421)

[Supplementary Figure 2. Prospective overall, liver related and cardiovascular mortality as a function of MLXIPL rs3812316 in the UKB subgroup of white participants and PMBB 7](#_Toc160546422)

[Supplementary Table 2. Multivariable PheWAS for MLXIPL rs3812316 in UKB corrected for age, sex, BMI, and PC1-10. 8](#_Toc160546423)

[Supplementary Table 3. Black heterozygous or homozygous carriers with non-carriers in UKB 11](#_Toc160546424)

[Supplementary Table 4. Characteristics of Black MLXIPL rs3812316 homozygous (A/A) or heterozygous (A/G) carriers compared with non-carriers (G/G) in UKB 12](#_Toc160546425)

[Supplementary Figure 3. Prospective overall, liver related and cardiovascular mortality as a function of MLXIPL rs35332062 and MLXIPL rs3812316 in the Black subgroup of the UKB 14](#_Toc160546426)

[Supplementary Table 6. Characteristics of Asian MLXIPL rs3812316 homozygous (A/A) or heterozygous (G/A) carriers compared with non-carriers (G/G) in UKB 16](#_Toc160546427)

[Supplementary Figure 4. Prospective overall, liver related and cardiovascular mortality as a function of MLXIPL rs35332062 and MLXIPL rs3812316 in the Asian subgroup of the UKB 18](#_Toc160546428)

[Supplementary Table 7: Characteristics of heterozygous MLXIPL rs1436362537 predicted loss of function variant compared with rs3812316 non-carriers in a UKB subgroup of white participants 19](#_Toc160546429)

[Supplementary Figure 5. Overview of the analyzed cohorts 20](#_Toc160546430)

[Supplementary Table 8. Characteristics of MLXIPL rs35332062 homozygous or heterozygous carriers compared with non-carriers in white British UKB participants 21](#_Toc160546431)

[Supplementary Table 9. Characteristics of MLXIPL rs35332062 homozygous or heterozygous carriers compared with non-carriers in white British UKB participants 22](#_Toc160546432)

[Supplementary Table 10. Baseline characteristics of homozygous (A/A) or heterozygous MLXIPL rs35332062 carriers (A/G) compared with non-carriers (G/G) in PMBB 24](#_Toc160546433)

[Supplementary Figure 6. Prospective overall, liver related and cardiovascular mortality as a function of MLXIPL rs35332062 in a UKB subgroup of white participants and PMBB 26](#_Toc160546434)

[Supplementary Table 11. Correlation analyses between MLXIPL rs35332062 and MLXIPL rs3812316 in UKB and PMBB 27](#_Toc160546435)

[Supplementary Figure 7. Associations of metabolic biomarkers of heterozygous MLXIPL rs139543215 loss of function variant for White UKB participants 28](#_Toc160546436)

[Supplementary Table 12: Cardiovascular Outcomes of MLXIPL rs3812316 homozygous (A/A) or heterozygous (G/A) carriers compared with non-carriers (G/G) in UKB 29](#_Toc160546437)

[Supplemental Table 13: Association between White carriers of the MLXIPL rs3812316 variant and BMI in the UKB 31](#_Toc160546438)

[Supplemental Table 14: Association between carriers of the MLXIPL rs3812316 variant and BMI in the PMBB 31](#_Toc160546439)

[Supplemental Table 15: Association between carriers of the MLXIPL rs3812316 variant and BMI ≥30kg/m^2^ in the UKB 32](#_Toc160546440)

[Supplemental Table 16: Association between carriers of the MLXIPL rs3812316 variant and BMI ≥30kg/m^2^ in the PMBB 32](#_Toc160546441)

## Supplementary Table 1. Definitions of Outcomes and Covariates

| Table 1. Definitions of Outcomes and Covariates | | |
| --- | --- | --- |
| Entity | **Source** | **Definition** |
| Age | UKB data field 21022 | Age at attending the Assessment center for the initial visit |
| Sex | UKB data field 31 | Sex of participant |
| BMI | UKB data field 21001 | BMI value is constructed from height and weight measured during the initial Assessment center visit |
| ALT | UKB data field 30620 | Alanine transaminase |
| AST | UKB data field 30650 | Aspartate transaminase |
| GGT | UKB data field 30730 | Gamma-glutamyl transferase |
| AP | UKB data field 30610 | Alkaline phosphatase |
| Triglycerides | UKB data field 30870 | Triglycerides |
| HDL | UKB data field 30760 | High-density lipoprotein |
| LDL | UKB data field 30780 | Low-density lipoprotein |
| Cholesterol | UKB data field 30690 | Cholesterol |
| Apolipoprotein A1 | UKB data field 30630 | Apolipoprotein A1 |
| Apolipoprotein B | UKB data field 30640 | Apolipoprotein B |
| HbA1c | UKB data field 30750 | Glycated hemoglobin |
| IGF-1 | UKB data field 30770 | Insulin-like growth factor 1 |
| Urate | UKB data field 30880 | Urate |
| Glucose | UKB data field 30740 | Glucose |
| ICD-10 codes | UKB data field 41270, extracted on 14th of April 2021 | Summary of the diagnosis codes a participant has recorded across all their hospital inpatient records |
| Diabetes mellitus | UKB data field 2443 | Baseline diabetes diagnosis by doctor |
| Ethnicity | UKB data field 21000 | Self-reported ethnicity |
| Alcohol drinker status | UKB data field 20117 | Self-reported alcohol consumption as “Never“, “Previous“ and “Current“ |
| Alcohol (g/d) | UKB data field 1558: Alcohol intake frequency UKB data field 407: Average monthly red wine intake UKB data field 4418: Average monthly champagne plus white wine intake  UKB data field 4429: Average monthly beer plus cider intake UKB data field 4440: Average monthly spirits intake  UKB data field 4451: Average monthly fortified wine intake UKB data field 4462: Average monthly intake of other alcoholic drinks  UKB data field 1568: Average weekly red wine intake UKB data field 1578: Average weekly champagne plus white wine intake  UKB data field 1588: Average weekly beer plus cider intake UKB data field 1598: Average weekly spirits intake  UKB data field 1608: Average weekly fortified wine intake UKB data field 5364: Average weekly intake of other alcoholic drinks | Estimated g/d alcohol consumption based on the alcohol intake frequency of alcohol consumption, the amount of beverage consumed as well as the amount of alcohol per standard pint of drink |
| Genetic principal components | UKB data field 22009 | Score for each principal component 1-40 |
| Survival | UKB data field 40000: date of death UKB data field 53: date of attending the assessment center  End of follow-up for those that did not die: 30/04/2021 | Date of death or end of Follow-up, Date of attending the Assessment Centre |
| Overall and cause specific mortality | UKB data field 40001, extracted on 14th of April 2021 | Primary cause of mortality during follow-up |

## Supplementary Figure 1. Multivariable PheWAS for *MLXIPL* rs3812316 in UKB corrected for age, sex, BMI, and PC1-10

Manhattan plot of adjusted −log10 (p-values) for all Phecodes comparing their occurrence. Highlighted are associations results with p-values <10-5. Upwards/downwards pointing trials refer to Phecodes that are over-/underrepresented.

**Abbreviations:** UKB, UK Biobank; BMI, body mass index; PC1-10, principal components of ancestry 1-10

Supplementary Figure 2. Prospective overall, liver related and cardiovascular mortality as a function of MLXIPL rs3812316 in the UKB subgroup of white participants and PMBB

Liver‐related mortality was defined as death due to liver diseases or hepatocellular carcinoma. **(A)** Overall mortality, **(B)** Liver‐related, **(C)** Cardiovascular mortality in the UKB population and **(D)** Overall mortality, **(E)** Liver‐related, **(F)** Cardiovascular mortality in the PMBB population as a function of *MLXIPL* *rs3812316* genotype. Participants were followed prospectively from the time of study entry until death or end of follow‐up. Hazard ratios were calculated by Cox regression, adjusted for age, sex, BMI, and PC1-4. Hazard ratios: **4A)** HR homozygotes vs. non-carrier: 0.97 [0.89-1.06]; HR heterozygotes vs. non-carrier: 1.02 [1-1.05] **4B)** HR homozygotes vs. non-carrier: 1.12 [0.70-1.79]; HR heterozygotes vs. non-carrier: 1.2 [1.04-1.39] **4C)** HR homozygotes vs. non-carrier: 0.93 [0.76-1.14]; HR heterozygotes vs. non-carrier: 1.07 [1.01-1.13]. **4D)** HR homozygotes vs. non-carrier: 1.19 [0.92-1.56]; HR heterozygotes vs. non-carrier: 1.01 [0.93-1.09] **4E)** HR homozygotes vs. non-carrier: 2.41 [0.58-9.98]; HR heterozygotes vs. non-carrier: 1.57 [0.94-2.64] **4F)** HR homozygotes vs. non-carrier: 0.85 [0.51-1.42]; HR heterozygotes vs. non-carrier: 1.01 [0.89-1.16].

**Abbreviations:** UKB, UK Biobank; PMBB, Penn Medicine BioBank; BMI, body mass index, PC1-10, principal components of ancestry 1-10; HR, hazard ratio

## Supplementary Table 2. Multivariable PheWAS for MLXIPL rs3812316 in UKB corrected for age, sex, BMI, and PC1-10.

| Phenotype | OR | p-value | Description |
| --- | --- | --- | --- |
| 274,1 | 0,810265 | 5,64E-12 | Gout |
| 274 | 0,820391 | 1,33E-11 | Gout and other crystal arthropathies |
| 272 | 0,964371 | 1,55E-04 | Disorders of lipoid metabolism |
| 272,1 | 0,964493 | 1,68E-04 | Hyperlipidemia |
| 250 | 1,047807 | 1,75E-04 | Diabetes mellitus |
| 701,6 | 1,615607 | 3,74E-04 | Acquired acanthosis nigricans |
| 250,2 | 1,045837 | 3,96E-04 | Type 2 diabetes |
| 735,21 | 0,866823 | 4,57E-04 | Hammer toe (acquired) |
| 272,11 | 0,966077 | 4,92E-04 | Hypercholesterolemia |
| 695,3 | 0,731226 | 5,02E-04 | Rosacea |
| 550,1 | 0,949014 | 6,00E-04 | Inguinal hernia |
| 507 | 1,060255 | 7,15E-04 | Pleurisy; pleural effusion |
| 696,2 | 2,765165 | 8,75E-04 | Parapsoriasis |
| 577,3 | 1,241792 | 1,25E-03 | Cyst and pseudocyst of pancreas |
| 743,4 | 10,2034 | 1,33E-03 | Stress fracture |
| 401 | 0,976126 | 2,01E-03 | Hypertension |
| 401,1 | 0,976253 | 2,14E-03 | Essential hypertension |
| 618 | 0,948337 | 2,51E-03 | Genital prolapse |
| 592,11 | 0,590032 | 0,00276 | Acute cystitis |
| 170,2 | 0,737275 | 0,003 | Cancer of connective tissue |
| 733,2 | 1,979696 | 0,00321 | Cyst of bone |
| 170 | 0,779956 | 0,004189 | Cancer of bone and connective tissue |
| 574,11 | 1,112731 | 0,005209 | Cholelithiasis with acute cholecystitis |
| 577 | 1,096303 | 0,00573 | Diseases of pancreas |
| 707 | 0,896575 | 0,006384 | Chronic ulcer of skin |
| 323,8 | 1,779499 | 0,006564 | Encephalitis, non-infectious |
| 703,1 | 0,831271 | 0,006926 | Ingrowing nail |
| 575 | 1,065386 | 0,007898 | Other biliary tract disease |
| 710,19 | 0,588687 | 0,008283 | Unspecified osteomyelitis |
| 726,2 | 1,24848 | 0,0086 | Synoviopathy |
| 333,4 | 1,257539 | 0,009905 | Torsion dystonia |
| 702 | 0,946491 | 0,009988 | Degenerative skin conditions and other dermatoses |
| 411,3 | 0,964325 | 0,011229 | Angina pectoris |
| 695,22 | 0,632388 | 0,011746 | Pemphigus and pemphigoid |
| 573,5 | 1,136691 | 0,012348 | Jaundice (not of newborn) |
| 817 | 0,424246 | 0,012513 | Concussion |
| 386,2 | 0,855454 | 0,013568 | Peripheral or central vertigo |
| 261 | 0,931704 | 0,013891 | Vitamin deficiency |
| 555,2 | 0,924752 | 0,014442 | Ulcerative colitis |
| 191,1 | 1,164581 | 0,015136 | Cancer of brain and nervous system |
| 286,1 | 1,202448 | 0,015684 | Congenital coagulation defects |
| 560 | 1,058369 | 0,016134 | Intestinal obstruction without mention of hernia |
| 386 | 0,916291 | 0,016174 | Vertiginous syndromes and other disorders of vestibular system |
| 871 | 0,940659 | 0,016433 | Open wounds of extremities |
| 611 | 0,891422 | 0,016832 | Abnormal findings on mammogram or breast exam |
| 292,2 | 0,827314 | 0,017114 | Mild cognitive impairment |
| 474,1 | 1,186227 | 0,017341 | Acute tonsillitis |
| 710,1 | 0,673986 | 0,017368 | Osteomyelitis |
| 710 | 0,680433 | 0,017416 | Osteomyelitis, periostitis, and other infections involving bone |
| 613,7 | 0,803918 | 0,017803 | Other signs and symptoms in breast |
| 611,3 | 0,890606 | 0,018122 | Lump or mass in breast |
| 729,3 | 1,589046 | 0,018221 | Panniculitis |
| 695,2 | 0,723024 | 0,018819 | Bullous dermatoses |
| 79,2 | 1,593794 | 0,019385 | Infectious mononucleosis |
| 579,8 | 0,925783 | 0,020353 | Nonspecific abnormal findings in stool contents |
| 550 | 0,97989 | 0,021137 | Abdominal hernia |
| 564,8 | 1,082029 | 0,021601 | Abnormal findings on exam of gastrointestinal tract/ abdominal area |
| 191,11 | 1,161435 | 0,021606 | Cancer of brain |
| 502 | 1,098869 | 0,022893 | Post inflammatory pulmonary fibrosis |
| 618,6 | 0,847961 | 0,023355 | Vaginal enterocele, congenital or acquired |
| 575,2 | 1,119952 | 0,023436 | Obstruction of bile duct |
| 560,3 | 1,117735 | 0,023843 | Peritoneal or intestinal adhesions |
| 411,4 | 0,971787 | 0,024266 | Coronary atherosclerosis |
| 286 | 1,128025 | 0,024727 | Coagulation defects |
| 283,2 | 1,97493 | 0,024935 | Non-autoimmune hemolytic anemias |
| 159 | 1,048163 | 0,025355 | Malignant neoplasm of other and ill-defined sites within the digestive organs and peritoneum |
| 564,9 | 0,971971 | 0,025487 | Personal history of diseases of digestive system |
| 569,2 | 1,159179 | 0,02658 | Gastrointestinal complications |
| 870,5 | 1,178477 | 0,026603 | Open wound of lip and mouth |
| 571 | 1,055703 | 0,027133 | Chronic liver disease and cirrhosis |
| 455 | 0,971357 | 0,027165 | Hemorrhoids |
| 172,1 | 0,933301 | 0,027404 | Melanomas of skin, dx or hx |
| 172,11 | 0,933301 | 0,027404 | Melanomas of skin |
| 785 | 1,020985 | 0,027554 | Abdominal pain |
| 741,3 | 0,82385 | 0,02757 | Difficulty in walking |
| 618,1 | 0,953463 | 0,027629 | Prolapse of vaginal walls |
| 571,8 | 1,10475 | 0,029028 | Liver abscess and sequelae of chronic liver disease |
| 575,6 | 1,173226 | 0,029867 | Cholesterolosis of gallbladder |
| 609,2 | 0,673277 | 0,030096 | Abnormal spermatozoa |
| 748 | 1,489857 | 0,030174 | Anomalies of respiratory system, congenital |
| 522,5 | 0,893801 | 0,030795 | Periapical abscess |
| 187,1 | 0,612287 | 0,031443 | Malignant neoplasm of unspecified male genital organ |
| 496,1 | 1,07238 | 0,032051 | Emphysema |
| 1013 | 1,181706 | 0,03319 | Asphyxia and hypoxemia |
| 210 | 0,880818 | 0,033922 | Benign neoplasm of lip, oral cavity, and pharynx |
| 702,2 | 0,939211 | 0,034087 | Seborrheic keratosis |
| 721,8 | 0,765921 | 0,034308 | Other allied disorders of spine |
| 1015 | 1,062563 | 0,036181 | Effects of other external cause |
| 741 | 0,899446 | 0,036842 | Symptoms and disorders of the joints |
| 215 | 0,891063 | 0,037397 | Other benign neoplasm of connective and other soft tissue |
| 244,1 | 1,09253 | 0,037866 | Secondary hypothyroidism |
| 558 | 0,96843 | 0,037927 | Noninfectious gastroenteritis |
| 740,9 | 0,961968 | 0,038028 | Osteoarthrosis NOS |
| 987 | 1,403888 | 0,038386 | Toxic effect of other gases, fumes, or vapors |
| 735,3 | 0,954898 | 0,042531 | Hallux valgus (Bunion) |
| 809 | 1,408225 | 0,043355 | Fracture of unspecified bones |
| 705,1 | 1,997842 | 0,043706 | Dyshidrosis |
| 657 | 4,749003 | 0,044433 | Infections specific to the perinatal period |
| 722,3 | 1,484937 | 0,045822 | Schmorl's nodes |
| 261,4 | 0,928139 | 0,045841 | Vitamin D deficiency |
| 594,1 | 0,941649 | 0,046558 | Calculus of kidney |
| 622,2 | 0,933118 | 0,047632 | Mucous polyp of cervix |
| 360,3 | 0,504795 | 0,047862 | Hypotony of eye |
| 291,1 | 0,710975 | 0,048229 | Transient mental disorders due to conditions classified elsewhere |
| 738 | 0,871616 | 0,048876 | Other acquired musculoskeletal deformity |
| 755,6 | 1,267217 | 0,048883 | Other congenital anomalies of lower limb, including pelvic girdle |
| 707,1 | 1,255565 | 0,049623 | Decubitus ulcer |

Manhattan plot of adjusted −log10 (p-values) for all Phecodes comparing their occurrence. Highlighted are associations results with p-values <10-30. Upwards/downwards pointing trials refer to Phecodes that are over-/underrepresented.

**Abbreviations:** UKB, UK Biobank; PMBB, Penn Medicine BioBank; BMI, body mass index, PC1-10, principal components of ancestry 1-10; OR, odds ratio; NOS, not otherwise specified

## Supplementary Table 3. Black heterozygous or homozygous carriers with non-carriers in UKB

|  | Non carriers  (G/G)  *n= 6 974* | Heterozygotes ( A/G )  *n=537* | Homozygotes (A/A)  *n= 21* | p-Value  G/G vs A/G | p-Value  G/G vs A/A |
| --- | --- | --- | --- | --- | --- |
| Characteristics |  |  |  | *Univ.* | *Univ.* |
| Age (years) | **51.92** ±8.07 | **51.77** ±7.98 | **53.43** ±9.03 | .67 | .39 |
| BMI (kg/m^2^) | **29.50** ±5.41 | **29.51** ±5.14 | **30.36** ±5.59 | .96 | .47 |
| Alcohol (g/d) | **3.36** ±6.22 | **3.59** ±6.18 | **2.02** ±4.38 | .41 | .32 |
| Diabetes mellitus (%) | **0.11** ±0.32 | **0.11** ±0.32 | **0.20** ±0.41 | .93 | .36 |
| Ethnicity (% black) | 100 | 100 | 100 |  |  |
|  | | | | | |
| Frequency of well-known  MASLD influencing genes* |  |  |  | *Univ.* | *Univ.* |
| HSD17B13 *rs72621367:TA* | **0.1** ±0.30 | **0.12** ±0.33 | **0.29** ±0.46 | .14 | .08 |
| PNPLA3 *rs738409:G* | **0.25** ±0.47 | **0.24** ±0.46 | **0.33** ±0.48 | .58 | .42 |
|  | | | | | |
| Liver status |  |  |  |  |  |
| ALT (U/l) | **22.21** ±13.62 | **22.02** ±11.54 | **24.72** ±19.14 | .76 | .40 |
| AST (U/l) | **27.17** ±13.55 | **25.97** ±8.68 | **26.26** ±11.82 | .05 | .76 |
| GGT (U/l) | **41.85** ±39.40 | **38.36** ±32.7 | **48.39** ±55.37 | **.024** | .45 |
| Bilirubin (mg/dl) | **0.52** ±0.28 | **0.53** ±0.28 | **0.49** ±0.26 | .56 | .61 |
| AP (U/l) | **84.11** ±26.41 | **81.82** ±23.12 | **96.95** ±46.30 | .06 | .22 |
|  | | | | | |
| Lipid metabolism |  |  |  |  |  |
| Triglycerides (mg/dl) | **108.85** ±66.37 | **107.08** ±62.83 | **122.12** ±76.99 | .76 | .36 |
| HDL cholesterol (mg/dl) | **55.68** ±14.31 | **53.75** ±13.15 | **52.59** ±11.99 | **.018** | .37 |
| LDL cholesterol (mg/dl) | **126.06** ±32.48 | **126.84** ±31.32 | **126.06** ±35.19 | .63 | .99 |
| Cholesterol (mg/dl) | **202.63** ±42.54 | **201.47** ±39.83 | **199.54** ±39.83 | .53 | .76 |
| Apolipoprotein A1 (g/l) | **1.5** ±0.26 | **1.47** ±0.24 | **1.43** ±0.2 | **.017** | .3 |
| Apolipoprotein B (g/l) | **0.96** ±0.24 | **0.96** ±0.23 | **0.99** ±0.25 | .89 | .56 |
|  | | | | | |
| Additional serum parameters |  |  |  |  |  |
| IGF-1 (nmol/L) | **22.42** ±6.18 | **23.01** ±7.18 | **22.92** ±7.57 | **.042** | .71 |
| Urate (umol/L) | **311.18** ±80.93 | **307.06** ±81.53 | **281.40** ±68.17 | .27 | .09 |

Quantitative measures are expressed as means and standard deviations or as relative frequencies (%). All multivariable analyses were adjusted for age, sex, BMI, and PC1-10. *(0=non carrier, 1=heterozygous, 2=homozygous)
**Abbreviations:** BMI, body mass index; ALT, alanine transaminase; AST, aspartate transaminase; GGT, gamma-glutamyl transferase; AP, alkaline phosphatase; LDL, low-density lipoprotein; HDL, high-density lipoprotein; IGF1, insuline-like growth factor

## Supplementary Table 4. Characteristics of Black *MLXIPL* rs3812316 homozygous (A/A) or heterozygous (A/G) carriers compared with non-carriers (G/G) in UKB

|  | Non carriers  (G/G)  *n=* 6 974 | Heterozygotes (A/G)  *n= 537* | Homozygotes (A/A)  *n= 21* | p-Value  G/G vs A/G | p-Value  G/G vs A/A | aOR  G/G vs A/G | aOR  G/G vs A/A |
| --- | --- | --- | --- | --- | --- | --- | --- |
| Liver status |  |  |  | *Univ.* | *Univ.* |  |  |
| ALT ≥ULN, N(%) | 322 (4.62) | 22 (4.1) | 3 (14.29) | .59 | **.048** | 0.885 | 3.220  [0.944-10.987] |
| AST ≥ULN, N(%) | 364 (5.22) | 15 (3.43) | 3 (14.29) | **.013** | .08 | 0.520  [0.308-0.878] | 2.812 |
|  | | | | | | | |
| ICD10 coded diagnoses |  |  |  |  |  |  |  |
| Alcoholic liver disease (K70), N(%) | 10 (0.14) | 0 | 0 | .38 | .86 | - | - |
| Toxic liver disease (K71), N(%) | 3 (0.04) | 0 | 1 (4.76) | .63 | **1.6854E-19** | - | 116.183  [11.586-1 165.033] |
| Hepatic failure (K72), N(%) | 15 (0.22) | 1 (0.19) | 0 | .89 | .83 | 0.866 | - |
| Chronic Hepatitis (K73), N(%) | 7 (0.10) | 1 (0.19) | 0 | .56 | .89 | 1.857 | - |
| Fibrosis and cirrhosis (K74), N(%) | 12 (0.17) | 0 | 0 | .34 | .85 | - | - |
| Inflammatory liver diseases (K75), N(%) | 22 (0.32) | 3 (0.56) | 0 | .35 | .8 | 1.775 | - |
| MASH (K75.8), N(%) | 2 (0.03) | 1 (0.19) | 0 | .08 | .94 | 6.504 | - |
| Other liver diseases (K76), N(%) | 134 (1.92) | 15 (2.79) | 1 (4.76) | .16 | .35 | 1.467 | 2.552 |
| MASLD (K76.0), N(%) | 74 (1.06) | 8 (1.49) | 1 (4.76) | .36 | .10 | 1.410 | 4.662 |
| Malignant neoplasm of the liver and/or bile duct (C22), N(%) | 5 (0.07) | 2 (0.37) | 0 | **.028** | .90 | 5.210  [1.009-26.919] | - |
|  | | | | | | | |
| Survival |  |  |  |  |  |  |  |
| All-cause mortality, N(%) | 298 (4.27) | 35 (6.52) | 0 | **.015** | .33 | 1.562  [1.088-2.243] | - |
| Liver related death, N(%) | 7 (0.10) | 0 | 0 | .46 | .89 | - | - |
| Cardiovascular death, N(%) | 66 (0.95) | 6 (1.12) | 0 | .66 | .65 | 1.209 | - |

Quantitative measures are expressed as number of participants (N) and as relative frequencies (%). All multivariable analyses were adjusted for age, sex, BMI, and PC1-10).

**Abbreviations:** ALT, alanine transaminase; AST, aspartate transaminase; ULN, upper limit of normal; MASLD, metabolic dysfunction-associated steatotic liver disease; MASH, metabolic dysfunction-associated steatohepatitis

## Supplementary Figure 3. Prospective overall, liver related and cardiovascular mortality as a function of *MLXIPL* rs35332062 and MLXIPL rs3812316 in the Black subgroup of the UKB

Liver‐related mortality was defined as death due to liver diseases or hepatocellular carcinoma. **(A)** Overall mortality, **(B)** Liver‐related, **(C)** Cardiovascular mortality in the UKB population as a function of *MLXIPL* rs35332062 genotype and **(D)** Overall mortality, **(E)** Liver‐related, **(F)** Cardiovascular mortality as a function of *MLXIPL* *rs3812316* genotype. Participants were followed prospectively from the time of study entry until death or end of follow‐up. Hazard ratios were calculated by Cox regression, adjusted for age, sex, BMI, and PC1-4. Hazard ratios: **5A)** HR homozygotes vs. non-carrier: 0.63 [0.09-4.54]; HR heterozygotes vs. non-carrier: 1.34 [0.99-1.81] **5B)** HR homozygotes vs. non-carrier: 0.000002; HR heterozygotes vs. non-carrier: 0.000002 **5C)** HR homozygotes vs. non-carrier: 0.000048 [2.7539E-224 – 8.303E+214]; HR heterozygotes vs. non-carrier: 1.2 [0.62-2.35] **5D)** HR homozygotes vs. non-carrier: 0.000114 [5.5255E-82 – 2.341E+73]; HR heterozygotes vs. non-carrier: 1.56 [1.1-2.22] **5E)** HR homozygotes vs. non-carrier: 0.000002; HR heterozygotes vs. non-carrier: 0.000002 **5F)** HR homozygotes vs. non-carrier: 0.000128 [3.2809E-127 – 4.983E+163]; HR heterozygotes vs. non-carrier: 1.23 [0.53-2.84].

**Abbreviations:** UKB, UK Biobank; PMBB, Penn Medicine BioBank; BMI, body mass index, PC1-10, principal components of ancestry 1-10; HR, hazard ratio

**Supplementary Table 5. Characteristics of Asian *MLXIPL* rs3812316 homozygous (A/A) or heterozygous (G/A) carriers compared with non-carriers (G/G) in UKB**

|  | Non carriers  (G/G)  *n= 9 152* | Heterozygotes ( A/G)  *n= 1 659* | Homozygotes (A/A)  *n= 93* | p-Value  G/G vs A/G | p-Value  G/G vs A/A |
| --- | --- | --- | --- | --- | --- |
| Characteristics |  |  |  | *Univ.* | *Univ.* |
| Age (years) | **53.23** ±8.30 | **53.06** ±8.62 | **54.55** ±9.03 | .47 | .16 |
| BMI (kg/m^2^) | **26.72** ±4.42 | **26.88** ±4.35 | 26.94 ±4.34 | .19 | .64 |
| Alcohol (g/d) | **2.72** ±5.91 | **2.55** ±5.58 | **3.06** ±7.79 | .28 | .59 |
| Diabetes mellitus (%) | **0.16** ±0.36 | **0.17** ±0.38 | **0.15** ±0.36 | .08 | .97 |
| Ethnicity (% Asian) | 100 | 100 | 100 |  |  |
|  | | | | | |
| Frequency of well-known  MASLD influencing genes* |  |  |  | *Univ.* | *Univ.* |
| HSD17B13 *rs72621367:TA* | **0.38** ±0.56 | **0.38** ±0.57 | **0.28** ±0.5 | .75 | .07 |
| PNPLA3 *rs738409:G* | **0.5** ±0.62 | **0.50** ±0.63 | **0.48** ±0.62 | .65 | .78 |
|  | | | | | |
| Liver status |  |  |  |  |  |
| ALT (U/l) | **24.25** ±14.54 | **24.61** ±14.46 | **22.86** ±11.68 | .36 | .37 |
| AST (U/l) | **26.35** ±10.91 | **26.03** ±9.39 | **25.24** ±9.39 | .27 | .34 |
| GGT (U/l) | **36.31** ±36.87 | **34.18** ±31.19 | **32.74** ±26.32 | **.016** | .36 |
| Bilirubin (mg/dl) | **0.52** ±0.25 | **0.52** ±0.25 | **0.52** ±0.24 | .65 | .78 |
| AP (U/l) | **87.01** ±25.00 | **86.48** ±24.88 | **87.6** ±21.3 | .44 | .82 |
|  | | | | | |
| Lipid metabolism |  |  |  |  |  |
| Triglycerides (mg/dl) | **173.45** ±104.42 | **161.95** ±92.04 | **152.21** ±75.22 | **.000004** | .06 |
| HDL cholesterol (mg/dl) | **49.88** ±13.15 | **49.5** ±13.15 | **51.43** ±13.15 | .09 | .36 |
| LDL cholesterol (mg/dl) | **129.93** ±32.87 | **129.54** ±32.1 | **132.64** ±33.64 | .79 | .39 |
| Cholesterol (mg/dl) | **206.88** ±43.31 | **204.95** ±42.15 | **209.59** ±44.86 | .11 | .57 |
| Apolipoprotein A1 (g/l) | **1.43** ±0.25 | **1.41** ±0.24 | **1.45** ±0.26 | **.002** | .56 |
| Apolipoprotein B (g/l) | **1.00** ±0.23 | **0.99** ±0.23 | **1.01** ±0.25 | .24 | .65 |
|  | | | | | |
| Additional serum parameters |  |  |  |  |  |
| Urate (umol/L) | **318.34** ±79.98 | **131.31** ±77.83 | **300.54** ±68.39 | **.021** | **.016** |

Quantitative measures are expressed as means and standard deviations or as relative frequencies (%). All multivariable analyses were adjusted for age, sex, BMI, and PC1-10. *(0=non carrier, 1=heterozygous, 2=homozygous).
**Abbreviations:** BMI, body mass index; ALT, alanine transaminase; AST, aspartate transaminase; GGT, gamma-glutamyl transferase; AP, alkaline phosphatase; LDL, low-density lipoprotein; HDL, high-density lipoprotein

## Supplementary Table 6. Characteristics of Asian *MLXIPL* rs3812316 homozygous (A/A) or heterozygous (G/A) carriers compared with non-carriers (G/G) in UKB

|  | Non carriers  (G/G)  *n= 9 152* | Heterozygotes (A/G)  *n= 1 659* | Homozygotes (A/A)  *n= 93* | p-Value  G/G vs A/G | p-Value  G/G vs A/A | aOR  G/G vs A/G | aOR  G/G vs A/A |
| --- | --- | --- | --- | --- | --- | --- | --- |
| Liver status |  |  |  | Univ. | Univ. |  |  |
| ALT ≥ULN, N(%) | 631 (6.89) | 114 (6.87) | 12 (12.90) | .92 | **.029** | 0.989 | 1.957  [1.060-3.613] |
| AST ≥ULN, N(%) | 393 (4.29) | 60 (3.62) | 4 (4.30) | .19 | .96 | 0.830 | 0.975 |
|  | | | | | | | |
| ICD10 coded diagnoses |  |  |  |  |  |  |  |
| Overall liver disease (K70-K76), N(%) |  |  |  |  |  |  |  |
| Alcoholic liver disease (K70), N(%) | 17 (0.19) | 9 (0.54) | 0 | **.006** | .68 | 2.931  [1.304-6.586] | - |
| Toxic liver disease (K71), N(%) | 5 (0.05) | 3 (0.18) | 0 | .08 | .82 | 3.314 | - |
| Hepatic failure (K72), N(%) | 23 (0.25) | 5 (0.30) | 0 | .71 | .63 | 1.200 | - |
| Chronic Hepatitis (K73), N(%) | 2 (0.02) | 0 | 0 | .55 | .89 | - | - |
| Fibrosis and cirrhosis (K74), N(%) | 24 (0.26) | 6 (0.36) | 0 | .48 | .62 | 1.381 | - |
| Inflammatory liver diseases (K75), N(%) | 28 (0.31) | 5 (0.30) | 0 | .98 | .59 | 0.985 | - |
| MASH (K75.8), N(%) | 8 (0.09) | 2 (0.12) | 0 | .68 | .78 | 1.380 | - |
| Other liver diseases (K76), N(%) | 190 (2.08) | 44 (2.65) | 2 (2.15) | .14 | .96 | 1.285 | 1.037 |
| MASLD (K76.0), N(%) | 131 (1.43) | 33 (1.99) | 2 (2.15) | .09 | .89 | 1.398 | 1.513 |
| Malignant neoplasm of the liver and/or bile duct (C22), N(%) | 13 (0.14) | 1 (0.06) | 0 | .39 | .72 | 0.424 | - |
|  | | | | | | | |
| Survival |  |  |  |  |  |  |  |
| All-cause mortality, N(%) | 422 (4.61) | 75 (4.52) | 9 (9.68) | .87 | **.021** | 0.980 | 2.216  [1.107-4.438] |
| Liver related death, N(%) | 9 (0.1) | 1 (0.06) | 0 | .64 | .76 | 0.613 | - |
| Cardiovascular death, N(%) | 125 (1.37) | 24 (1.45) | 3 (3.23) | .8 | .13 | 1.060 | 2.407 |

Quantitative measures are expressed as number of participants (N) and as relative frequencies (%). All multivariable analyses were adjusted for age, sex, BMI, and PC1-10)

**Abbreviations:** ALT, alanine transaminase; AST, aspartate transaminase; ULN, upper limit of normal; MASLD, metabolic dysfunction-associated steatotic liver disease; MASH, metabolic dysfunction-associated steatohepatitis

## Supplementary Figure 4. Prospective overall, liver related and cardiovascular mortality as a function of *MLXIPL* rs35332062 and MLXIPL rs3812316 in the Asian subgroup of the UKB

Liver‐related mortality was defined as death due to liver diseases or hepatocellular carcinoma. **(A)** Overall mortality, **(B)** Liver‐related, **(C)** Cardiovascular mortality in the UKB population as a function of *MLXIPL* *rs35332062* genotype and **(D)** Overall mortality, **(E)** Liver‐related, **(F)** Cardiovascular mortality as a function of *MLXIPL* *rs3812316* genotype. Participants were followed prospectively from the time of study entry until death or end of follow‐up. Hazard ratios were calculated by Cox regression, adjusted for age, sex, BMI, and PC1-4. Hazard ratios: **6A)** HR homozygotes vs. non-carrier: 1.99 [1.03-3.87]; HR heterozygotes vs. non-carrier: 0.97 [0.75-1.24] **6B)** HR homozygotes vs. non-carrier: 0.000037; HR heterozygotes vs. non-carrier: 0.55 [0.07-4.63] **6C)** HR homozygotes vs. non-carrier: 2.42 [0.76-7.66|; HR heterozygotes vs. non-carrier: 1.11 [0.71-1.72] **6D)** HR homozygotes vs. non-carrier: 2.03 [1.04-3.94]; HR heterozygotes vs. non-carrier: 1 [0.78-1.28] **6E)** HR homozygotes vs. non-carrier: 0.000035; HR heterozygotes vs. non-carrier: 0.56 [0.07-4.71] **6F)** HR homozygotes vs. non-carrier: 2.45 [0.77-7.78]; HR heterozygotes vs. non-carrier: 1.12 [0.72-1.75].

**Abbreviations:** UKB, UK Biobank; PMBB, Penn Medicine BioBank; BMI, body mass index, PC1-10, principal components of ancestry 1-10; HR, hazard ratio

## Supplementary Table 7: Characteristics of heterozygous *MLXIPL* rs1436362537 predicted loss of function variant compared with rs3812316 non-carriers in a UKB subgroup of white participants

|  | **Non carriers**  **(G/G)**  *n= 200 639* | **Heterozygotes (A/G)**  *n= 4* | **p-Value**  **G/G vs A/G** |
| --- | --- | --- | --- |
| **Characteristics** |  |  | *Univ.* |
| Age (years) | **56.72** ±8.03 | **56.50** ±4.63 | 0.96 |
| BMI (kg/m^2^) | **27.34** ±4.73 | **23.68** ±2.39 | 0.12 |
| Alcohol (g/d) | **9.09** ±10.05 | **5.21** | 0.44 |
| Diabetes mellitus (%) | **0.05** ±5.27 | **0.0** ±0.0 | 0.66 |
| Ethnicity (% White) | 100 | 100 |  |
|  | | | |
|  | | | |
| **Liver profile** |  |  |  |
| ALT (U/l) | **23.37** ±13.73 | **16.54** ±2.52 | 0.32 |
| AST (U/l) | **26.07** ±9.98 | **21.08** ±2.03 | 0.32 |
| GGT (U/l) | **36.04** ±40.29 | **17.73** ±1.83 | 0.35 |
| AP (U/l) | **83.32** ±25.66 | **78.30** ±26.92 | 0.7 |
|  | | | |
| **Lipid profile** |  |  |  |
| Triglycerides (mg/dl) | **152.25** ±88.38 | **119** ±74.13 | 0.46 |
| HDL cholesterol (mg/dl) | **56.46** ±14.69 | **64.19** ±17.01 | 0.36 |
| LDL cholesterol (mg/dl) | **138.05** ±33.26 | **164.73** ±11.99 | 0.11 |
| Cholesterol (mg/dl) | **221.19** ±43.7 | **255.22** ±22.04 | 0.12 |
| Apolipoprotein A1 (g/l) | **1.55** ±0.27 | **1.62** ±0.23 | 0.64 |
| Apolipoprotein B (g/l) | **1.03** ±0.24 | **1.19** ±0.04 | **0.004** |
|  | | | |
| Urate (umol/L) | **307.67** ±79.82 | **263.53** ±57.84 | 0.27 |
|  | | | |
| **ICD10 coded diagnoses** |  |  |  |
| Alcoholic liver disease (K70), N(%) | 488 (0.24) | 0 | 0.92 |
| Toxic liver disease (K71), N(%) | 45 (0.02) | 0 | 0.98 |
| Hepatic failure (K72), N(%) | 329 (0.16) | 0 | 0.93 |
| Chronic Hepatitis (K73), N(%) | 78 (0.04) | 0 | 0.97 |
| Fibrosis and cirrhosis (K74), N(%) | 667 (0.33) | 0 | 0.91 |
| Inflammatory liver diseases (K75), N(%) | 599 (0.3) | 0 | 0.91 |
| NASH (K75.8), N(%) | 215 (0.11) | 0 | 0.95 |
| NAFLD (K76.0), N(%) | 1 975 (0.98) | 0 | 0.84 |

Quantitative measures are expressed as means and standard deviations or as relative frequencies (%). All multivariable analyses were adjusted for age, sex, BMI, and PC1-4.

**Abbreviations:** BMI, body mass index; ALT, alanine transaminase; AST, aspartate transaminase; GGT, gamma-glutamyl transferase; AP, alkaline phosphatase; LDL, low-density lipoprotein; HDL, high-density lipoprotein; NAFLD, non-alcoholic fatty liver disease; NASH, non-alcoholic steatohepatitis

## Supplementary Figure 5. Overview of the analyzed cohorts

1. UK Biobank participants of European ancestry aged 37 to 73 years (*MLXIP*L rs35332062)
2. Penn Medicine Biobank (PMBB) participants of 55% African American ancestry aged 25 to 105 years (*MLXIPL* rs35332062)

## Supplementary Table 8. Characteristics of MLXIPL rs35332062 homozygous or heterozygous carriers compared with non-carriers in white British UKB participants

|  | Non carriers  (G/G)  *n= 246 390* | Heterozygotes (A/G)  *n= 101 001* | Homozygotes (A/A)  *n= 7 451* | p-Value  G/G vs A/G | p-Value  G/G vs A/A |
| --- | --- | --- | --- | --- | --- |
| Characteristics |  |  |  | *Univ.* | *Univ.* |
| Age (years) | **56.78** ±8.03 | **56.79** ±8.02 | **56.70** ±8.05 | .79 | .40 |
| BMI (kg/m^2^) | **27.38** ±4.76 | **27.44** ±4.78 | 27.55 ±4.91 | **.000171** | **.002** |
| Alcohol (g/d) | **9.04** ±10.12 | **9.25** ±10.28 | **9.55** ±10.42 | **8E-9** | **.000033** |
| Diabetes mellitus (%) | **0.05** ±0.21 | **0.05** ±0.22 | **0.05** ±0.22 | .07 | .31 |
| Ethnicity (% white) | 100 | 100 | 100 |  |  |
|  | | | | | |
| Frequency of well-known  MASLD influencing genes* |  |  |  | *Multiv.* | *Multiv.* |
| HSD17B13 *rs72621367:TA* | **0.55** ±0.63 | **0.55** ±0.63 | 0.55 ±0.63 | .49 | .94 |
| PNPLA3 *rs738409:G* | **0.43** ±0.58 | **0.43** ±0.58 | 0.43 ±0.58 | .56 | .35 |
|  | | | | | |
| Liver status |  |  |  |  |  |
| ALT (U/l) | **23.36** ±13.80 | **23.81** ±14.48 | **24.08** ±14.63 | **1.2E-17** | **.000034** |
| AST (U/l) | **26.13** ±10.04 | **26.13** ±11.15 | **25.89** ±9.74 | 1.000 | **.042** |
| GGT (U/l) | **37.30** ±41.34 | **35.91** ±39.90 | **34.22** ±40.63 | **4.8E-21** | **2.6E-10** |
| Bilirubin (mg/dl) | **0.53** ±0.26 | **0.53** ±0.26 | **0.54** ±0.28 | .23 | .011 |
| AP (U/l) | **83.76** ±26.48 | **83.02** ±25.97 | **81.79** ±24.03 | **5.8E-15** | **8.3E-12** |
|  | | | | | |
| Lipid metabolism |  |  |  |  |  |
| Triglycerides (mg/dl) | **157.52** ±92.04 | **146.9** ±84.96 | **135.4** ±74.34 | **8.7E-251** | **2.2E-128** |
| HDL cholesterol (mg/dl) | **56.07** ±14.69 | **56.46** ±14.69 | **57.23** ±15.08 | **9.5E-20** | **1.3E-11** |
| LDL cholesterol (mg/dl) | **138.05** ±33.64 | **138.05** ±33.64 | **138.05** ±33.26 | .91 | .85 |
| Cholesterol (mg/dl) | **220.81** ±44.08 | **220.42** ±44.08 | **220.42** ±43.7 | **.012** | .27 |
| Apolipoprotein A1 (g/l) | **1.54** ±0.27 | **1.54** ±0.27 | **1.54** ±0.27 | .26 | .80 |
| Apolipoprotein B (g/l) | **1.04** ±0.24 | **1.03** ±0.24 | **1.02** ±0.23 | **5.4E-10** | **.000055** |
|  | | | | | |
| Additional serum parameters |  |  |  |  |  |
| HbA1c  (mmol/mol) | **35.94** ±6.47 | **36.05** ±6.69 | **36.1** ±6.48 | **.000006** | **.045** |
| IGF-1 (nmol/L) | **21.37** ±5.67 | **21.52** ±5.67 | **21.76** ±5.86 | **4.6E-13** | **2.9E-8** |
| Urate (umol/L) | **309.6** ±80.48 | **306.47** ±79.56 | **301.34** ±77.72 | **1.1E-26** | **1.0E-18** |

Quantitative measures are expressed as means and standard deviations or as relative frequencies (%). All multivariable analyses were adjusted for age, sex, BMI, and PC1-10. *(0=non carrier, 1=heterozygous, 2=homozygous).

**Abbreviations:** BMI, body mass index; ALT, alanine transaminase; AST, aspartate transaminase; GGT, gamma-glutamyl transferase; AP, alkaline phosphatase; LDL, low-density lipoprotein; HDL, high-density lipoprotein; HbA1c, Hemoglobin A1; IGF1, insuline-like growth factor

## Supplementary Table 9. Characteristics of *MLXIPL* rs35332062 homozygous or heterozygous carriers compared with non-carriers in white British UKB participants

|  | Non carriers  (G/G)  *n= 246 390* | Heterozygotes (A/G)  *n= 101 001* | Homozygotes (A/A)  *n= 7 451* | p-Value  G/G vs A/G | p-Value  G/G vs A/A | aOR  G/G vs A/G | aOR  G/G vs A/A |
| --- | --- | --- | --- | --- | --- | --- | --- |
| Liver status |  |  |  | Multiv. | Multiv. |  |  |
| ALT ≥ULN, N(%) | 21 815 (6.3) | 6 990 (6.92) | 550 (7.38) | **7.3876E-13** | **.000158** | 1.108  [1.077-1.139] | 1.185  [1.058-1.294] |
| AST ≥ULN, N(%) | 15 352 (4.43) | 4 406 (4.36) | 319 (4.28) | .37 | .51 | 0.984 | 0.962 |
|  | | | | | | | |
| ICD10 coded diagnoses |  |  |  |  |  |  |  |
| Overall liver disease (K70-K76), N(%) |  |  |  |  |  |  |  |
| Alcoholic liver disease (K70), N(%) | 1 047 (0.30) | 317 (0.31) | 30 (0.40) | .56 | .12 | 1.038 | 1.333 |
| Toxic liver disease (K71), N(%) | 104 (0.03) | 19 (0.02) | 2 (0.03) | .06 | .88 | 0.626 | 0.894 |
| Hepatic failure (K72), N(%) | 587 (0.17) | 195 (0.19) | 19 (0.25) | .11 | .08 | 1.140 | 1.506 |
| Chronic Hepatitis (K73), N(%) | 144 (0.04) | 36 (0.04) | 3 (0.04) | .41 | .96 | 0.857 | 0.969 |
| Fibrosis and cirrhosis (K74), N(%) | 1 367 (0.39) | 368 (0.36) | 27 (0.36) | .17 | .66 | 0.923 | 0.918 |
| Inflammatory liver diseases (K75), N(%) | 1 105 (0.32) | 351 (0.35) | 21 (0.28) | .16 | .57 | 1.090 | 0.883 |
| MASH (K75.8), N(%) | 406 (0.12) | 124 (0.12) | 7 (0.09) | .65 | .56 | 1.048 | 0.801 |
| Other liver diseases (K76), N(%) | 6 954 (2.01) | 2 129 (2.11) | 169 (2.27) | **.047** | .11 | 1.051 [1.001-1.104] | 1.133 |
| MASLD (K76.0), N(%) | 3 748 (1.08) | 1 115 (1.01) | 104 (1.4) | .55 | **.010** | 1.020 | 1.294  [1.064-1.575] |
| Malignant neoplasm of the liver and/or bile duct (C22), N(%) | 638 (0.18) | 189 (0.19) | 10 (0.13) | .85 | .32 | 1.016 | 0.728 |
|  | | | | | | | |
| Survival |  |  |  |  |  |  |  |
| All-cause mortality, N(%) | 23 961 (6.92) | 7 210 (0.07) | 503 (6.75) | **.015** | .58 | 1.034  [1.007-1.063] | 0.974 |
| Liver related death, N(%) | 719 (0.21) | 248 (0.25) | 18 (0.24) | **.022** | .52 | 1.183  [1.024-1.367] | 1.164 |
| Cardiovascular death, N(%) | 4 801 (1.39) | 1 510 (1.5) | 99 (1.33) | **.010** | .68 | 1.080  [1.019-1.145] | 0.958 |

Quantitative measures are expressed as number of participants (N) and as relative frequencies (%). All multivariable analyses were adjusted for age, sex, BMI, and PC1-10).

**Abbreviations:** ALT, alanine transaminase; AST, aspartate transaminase; ULN, upper limit of normal; MASLD, metabolic dysfunction-associated steatotic liver disease; MASH, metabolic dysfunction-associated steatohepatitis

## Supplementary Table 10. Baseline characteristics of homozygous (A/A) or heterozygous *MLXIPL* rs35332062 carriers (A/G) compared with non-carriers (G/G) in PMBB

|  | Non carriers (G/G)  *n= 32 466* | Heterozygotes (G/A)  *n= 7 706* | Homozygotes (A/A)  *n= 468* | p-Value  G/G vs G/A | p-Value  G/G vs A/A |
| --- | --- | --- | --- | --- | --- |
| Characteristics |  |  |  | *Univ.* | *Univ.* |
| Age (years) | **54.95** ±16.75 | **55.91** ±16.54 | **56.73** ±16.71 | **.000005** | **.022** |
| Women (%) | 35.9 | 35.9 | 35.9 |  |  |
| BMI (kg/m^2^) | **29.50** ±7.27 | **28.97** ±6.75 | **29.04** ±7.02 | **5.9985E-9** | .20 |
| Diabetes mellitus (%) | **0.25** ±0.43 | **0.25** ±0.43 | **0.26** ±0.44 | .87 | .87 |
| Ethnicity (% Whites) | 47.9 | 47.9 | 47.9 |  |  |
|  | | | | | |
| Frequency of well-known MASLD influencing genes* | | | |  |  |
| HSD17B13 *rs72621367:TA* | **0.29** ±0.52 | **0.38** ±0.57 | **0.48** ±0.63 | **9.596E-12** | **.001** |
| PNPLA3 *rs738409:G* | **0.43** ±0.59 | **0.43** ±0.59 | **0.45** ±0.6 | .89 | .54 |
|  | | | | | |
| Liver status |  |  |  | *Multiv.* | *Multiv.* |
| ALT (U/l) | **24.58** ±42.18 | **25.50** ±47.04 | **30.26** ±56.28 | .13 | .05 |
| ALT ≥ULN (%) | 1 236 (3.81) | 297 (3.85) | 25 (5.34) | .85 | .09 |
| AST (U/l) | **24.77** ±39.79 | **25.06** ±37.66 | **29.05** ±38.17 | .6 | **.030** |
| AST ≥ULN (%) | 912 (2.81) | 197 (2.56) | 23 (4.91) | .22 | **.006** |
|  |  |  |  |  |  |
| Lipid metabolism |  |  |  |  |  |
| Triglycerides (mg/dl) | **122.51** ±87.22 | **118.49** ±86.83 | **112.00** ±64.16 | **.006** | .05 |
| HDL cholesterol (mg/dl) | **50.25** ±15.38 | **50.28** ±15.73 | **48.74** ±16.17 | .94 | .41 |
| LDL cholesterol (mg/dl) | **99.15** ±32.64 | **98.87** ±32.21 | **98.99** ±32.54 | .61 | .94 |
| Cholesterol (mg/dl) | **175.26** ±39.67 | **174.36** ±39.28 | **173.03** ±38.31 | .18 | .37 |
|  | | | | | |
| ICD10 coded diagnoses |  |  |  |  |  |
| Toxic liver disease (K71) | 209 (0.64) | 31 (0.40) | 2 (0.43) | **.013**^a^ | .56 |
| Hepatic failure (K72) | 208 (0.64) | 45 (0.58) | 7 (1.5) | .57 | **.023**^b^ |
| Chronic Hepatitis (K73) | 22 (0.07) | 3 (0.04) | 0 | .36 | .57 |
| Fibrosis and cirrhosis (K74) | 156 (0.48) | 48 (0.62) | 3 (0.64) | .11 | .62 |
| Inflammatory liver diseases (K75) | 653 (2.01) | 146 (1.89) | 9 (1.92) | .51 | .89 |
| MASH (K75.8) | 742 (2.29) | 159 (2.06) | 9 (1.92) | .24 | .60 |
| Other liver diseases (K76) | 1 418 (4.37) | 344 (4.46) | 30 (6.41) | .71 | .06 |
| MASLD (K76.0) | 1 086 (3.35) | 251 (3.26) | 22 (4.70) | .7 | .11 |

Quantitative measures are expressed as means and standard deviations or as relative frequencies (%). All multivariable analyses were adjusted for age, sex, BMI, and PC1-4. *(0=non carrier, 1=heterozygous, 2=homozygous); ^a^aOR=0.623[0.427-0.91]; ^b^aOR=2.355[0.1.103-5.029].

**Abbreviations:** BMI, body mass index; ALT, alanine transaminase; AST, aspartate transaminase; ULN, upper limit of normal; GGT, gamma-glutamyl transferase; HDL, high-density lipoprotein; LDL, low-density lipoprotein; MASLD, metabolic dysfunction-associated steatotic liver disease; MASH, metabolic dysfunction-associated steatohepatitis

## Supplementary Figure 6. Prospective overall, liver related and cardiovascular mortality as a function of *MLXIPL* rs35332062 in a UKB subgroup of white participants and PMBB

Liver‐related mortality was defined as death due to liver diseases or hepatocellular carcinoma. **(A)** Overall mortality, **(B)** Liver‐related, **(C)** Cardiovascular mortality in the UKB population and **(D)** Overall mortality, **(E)** Liver‐related, **(F)** Cardiovascular mortality in PMBB as a function of *MLXIPL* *rs35332062* genotype. Participants were followed prospectively from the time of study entry until death or end of follow‐up. Hazard ratios were calculated by Cox regression, adjusted for age, sex, BMI, and PC1-4. Hazard ratios: **4A)** HR homozygotes vs. non-carrier: 0.96 [0.88-1.05]; HR heterozygotes vs. non-carrier: 1.02 [1-1.05] **4B)** HR homozygotes vs. non-carrier: 1.13 [0.71-1.80]; HR heterozygotes vs. non-carrier: 1.17 [1.01-1.35] **4C)** HR homozygotes vs. non-carrier: 0.94 [0.77-1.15]; HR heterozygotes vs. non-carrier: 1.07 [1.00-1.13] **4D)** HR homozygotes vs. non-carrier: 1.24 [0.96-1.61]; HR heterozygotes vs. non-carrier: 0.99 [0.92-1.08] **4E)** HR homozygotes vs. non-carrier: 2.42 [0.59-9.99]; HR heterozygotes vs. non-carrier: 1.63 [0.99-2.69] **4F)** HR homozygotes vs. non-carrier: 1.02 [0.64-1.63]; HR heterozygotes vs. non-carrier: 0.99 [0.87-1.1].

**Abbreviations:** UKB, UK Biobank; PMBB, Penn Medicine BioBank; BMI, body mass index, PC1-10, principal components of ancestry 1-10; HR, hazard ratio

## Supplementary Table 11. Correlation analyses between MLXIPL rs35332062 and MLXIPL rs3812316 in UKB and PMBB

|  |  | Pearson Correlation | p-value | LD |
| --- | --- | --- | --- | --- |
| UKB White | | | |  |
|  | *MLXIPL* rs35332062 | 0.983 | .0e0 |  |
|  | *MLXIPL* rs3812316 | 0.983 | .0e0 |  |
|  |  |  |  | 0.965 |
| UKB Black | | | |  |
|  | *MLXIPL* rs35332062 | 0.711 | .0e0 |  |
|  | *MLXIPL* rs3812316 | 0.711 | .0e0 |  |
|  |  |  |  | 0.501 |
| UKB Asian | | | |  |
|  | *MLXIPL* rs35332062 | 0.978 | .0e0 |  |
|  | *MLXIPL* rs3812316 | 0.978 | .0e0 |  |
|  |  |  |  | 0.955 |
| PMBB | | | |  |
|  | *MLXIPL* rs35332062 | 0.951 | .0e0 |  |
|  | *MLXIPL* rs3812316 | 0.951 | .0e0 |  |

**Abbreviations:** UKB, UK Biobank; PMBB, Penn Medicine BioBank; LD, linkage disequilibrium


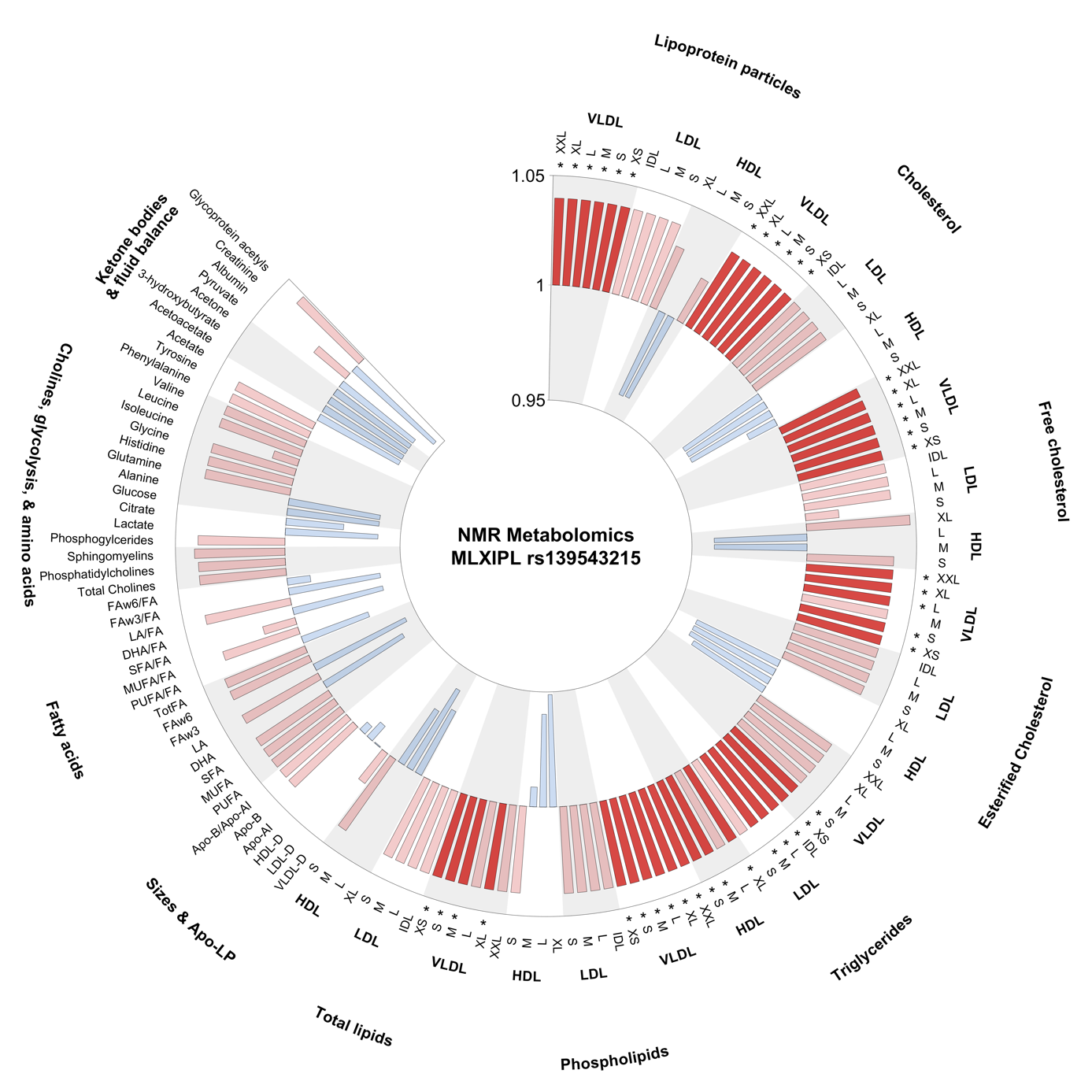


## Supplementary Figure 7. Associations of metabolic biomarkers of heterozygous *MLXIPL* rs139543215 loss of function variant for White UKB participants

Hazard ratios (with 95% confidence intervals) are presented per 1-SD higher metabolic biomarker on the natural log scale, stratified by age, sex, body mass index and PC1-10. *False discovery rate-controlled p < 0.01. Positive associations are displayed in red while negative associations are blue.

**Abbreviations:** DHA, docosahexaenoic acid; FA, fatty acids; FAw3, omega-3 fatty acids; FAw6, omega-6 fatty acids; HDL-D, high-density lipoprotein particle diameter; LA, linoleic acid; LDL, low-density lipoproteins; LDL-D, low-density lipoprotein particle diameter; LP, lipoprotein; MUFA, monounsaturated fatty acids; PUFA, polyunsaturated fatty acids; SFA, saturated fatty acids; VLDL-D, very low-density lipoprotein particle diameter; (original code by Diego J Aguilar-Ramirez).

## Supplementary Table 12: Cardiovascular Outcomes of *MLXIPL* rs3812316 homozygous (A/A) or heterozygous (G/A) carriers compared with non-carriers (G/G) in UKB

|  | Non carriers  (G/G)  *n=* | Heterozygotes (A/G)  *n=* | Homozygotes (A/A)  *n=* | p-Value  G/G vs A/G | p-Value  G/G vs A/A | aOR  G/G vs. A/G | aOR  G/G vs. A/A |
| --- | --- | --- | --- | --- | --- | --- | --- |
|  | | | | *Univ.* | *Univ.* |  | |
| ICD10 coded diagnoses | | | |  |  |  | |
| Hypertensive heart disease (I11), N (%) | 231 | 66 | 2 | .86 | .18 |  |  |
| Hypertensive heart and chronic kidney disease (I13), N (%) | 74 | 23 | 2 | .81 | .76 |  |  |
| Secondary Hypertension (I15), N (%) | 157 | 44 | 1 | .79 | .19 |  |  |
| Angina pectoris (I20), N (%) | 21 393 | 6 187 | 417 | .35 | **.019** |  | 0.887  [0.803-0.980] |
| Acute myocardial infarction (I21), N (%) | 10 523 | 3 200 | 207 | .06 | .13 |  |  |
| Subsequent ST elevation (STEMI) and non-ST elevation (NSTEMI) myocardial infarction (I22), N (%) | 643 | 190 | 13 | .92 | .79 |  |  |
| Certain current complications following ST elevation (STEMI) and non-ST elevation (NSTEMI) myocardial infarction (I23), N (%) | 66 | 14 | 3 | .27 | .20 |  |  |
| Other acute ischemic heart disease (I24), N (%) | 2 608 | 758 | 63 | .84 | .42 |  |  |
| Chronic ischemic heart disease (I25), N (%) | 32 502 | 9 420 | 668 | .33 | .11 |  |  |
| Cardiomyopathy (I42), N (%) | 1 994 | 610 | 37 | .35 | .33 |  |  |
| Cardiac arrest(I46), N (%) | 1 781 | 518 | 38 | .88 | .89 |  |  |
| Heart failure (I50), N (%) | 10 407 | 3 117 | 221 | .28 | .69 |  |  |
| Atherosclerosis (I70), N (%) | 812 | 248 | 12 | .57 | .18 |  |  |
| Arterial embolism and thrombosis (I74), N (%) | 1 220 | 376 | 33 | .39 | .22 |  |  |

Quantitative measures are expressed as number of participants (N) and as relative frequencies (%). All multivariable analyses were adjusted for age, sex, BMI, and PC1-10).

**Abbreviations:** UKB, UK Biobank

## Supplemental Table 13: Association between White carriers of the *MLXIPL* rs3812316 variant and BMI in the UKB

|  | Sig. |
| --- | --- |
| *MLXIPL* rs3812316 | .097 |
| *MLXIPL* rs3812316 and BMI | **2.4959E-14** |
| Age | **2.8397E-7** |
| BMI | 0.0 |
| Sex | **.000024** |
| Genetic principal components 1 | .24 |
| Genetic principal components 2 | **.04** |
| Genetic principal components 3 | .38 |
| Genetic principal components 4 | .84 |
| Genetic principal components 5 | **.006** |
| Genetic principal components 6 | **.001** |
| Genetic principal components 7 | .88 |
| Genetic principal components 8 | .004 |
| Genetic principal components 9 | **.000098** |
| Genetic principal components 10 | .11 |

## Supplemental Table 14: Association between carriers of the *MLXIPL* rs3812316 variant and BMI in the PMBB

|  | Sig. |
| --- | --- |
| *MLXIPL* rs3812316 | .51 |
| *MLXIPL* rs3812316 and BMI | **.000481** |
| Age | .66 |
| BMI | **7.5245E-160** |
| Sex | .22 |
| Genetic principal components 1 | .82 |
| Genetic principal components 2 | .75 |
| Genetic principal components 3 | .68 |
| Genetic principal components 4 | .08 |
| Genetic principal components 5 | .07 |
| Genetic principal components 6 | .87 |
| Genetic principal components 7 | .37 |
| Genetic principal components 8 | .12 |
| Genetic principal components 9 | .48 |
| Genetic principal components 10 | .93 |

**Abbreviations:** UKB, UK Biobank; BMI, body mass index; PMBB, Penn Medicine BioBank

## Supplemental Table 15: Association between carriers of the *MLXIPL* rs3812316 variant and BMI ≥30kg/m^2^ in the UKB

|  | Sig. |
| --- | --- |
| *MLXIPL* rs3812316 | **7.8755E-9** |
| *MLXIPL* rs3812316 and BMI ≥30kg/m^2^ | **2.4636E-7** |
| Age | **.000007** |
| BMI | 0.0 |
| Sex | **.002** |
| Genetic principal components 1 | .93 |
| Genetic principal components 2 | .71 |
| Genetic principal components 3 | .83 |
| Genetic principal components 4 | .32 |
| Genetic principal components 5 | .1 |
| Genetic principal components 6 | **.048** |
| Genetic principal components 7 | .86 |
| Genetic principal components 8 | **.043** |
| Genetic principal components 9 | **.000004** |
| Genetic principal components 10 | .75 |

## Supplemental Table 16: Association between carriers of the *MLXIPL* rs3812316 variant and BMI ≥30kg/m^2^ in the PMBB

|  | Sig. |
| --- | --- |
| *MLXIPL* rs3812316 | **.002** |
| *MLXIPL* rs3812316 and BMI ≥30kg/m^2^ | .012 |
| Age | .46 |
| BMI | **9.3167E-119** |
| Sex | .69 |
| Genetic principal components 1 | **.008** |
| Genetic principal components 2 | .57 |
| Genetic principal components 3 | .66 |
| Genetic principal components 4 | .34 |
| Genetic principal components 5 | .25 |
| Genetic principal components 6 | .64 |
| Genetic principal components 7 | .96 |
| Genetic principal components 8 | .29 |
| Genetic principal components 9 | .59 |
| Genetic principal components 10 | 7 |
